# Local Intrinsic Dimensionality, Entropy and Statistical Divergences

**DOI:** 10.3390/e24091220

**Published:** 2022-08-30

**Authors:** James Bailey, Michael E. Houle, Xingjun Ma

**Affiliations:** 1 School of Computing and Information Systems, The University of Melbourne, Melbourne, VIC 3010, Australia; 2 School of Computer Science, Fudan University, Shanghai 200437, China

**Keywords:** entropy, tail entropy, cumulative entropy, entropy power, intrinsic dimensionality, local intrinsic dimension, statistical divergences, statistical distances

## Abstract

Properties of data distributions can be assessed at both global and local scales. At a highly localized scale, a fundamental measure is the local intrinsic dimensionality (LID), which assesses growth rates of the cumulative distribution function within a restricted neighborhood and characterizes properties of the geometry of a local neighborhood. In this paper, we explore the connection of LID to other well known measures for complexity assessment and comparison, namely, entropy and statistical distances or divergences. In an asymptotic context, we develop analytical new expressions for these quantities in terms of LID. This reveals the fundamental nature of LID as a building block for characterizing and comparing data distributions, opening the door to new methods for distributional analysis at a local scale.

## 1. Introduction

Fundamental activities for analyzing data include both an ability to characterize data complexity and an ability to make comparisons between distributions. Widely used measures for these activities include entropy (for assessing uncertainty) and statistical divergences or distances (to compare distributions) [1]. Such analysis can be performed at either a global scale across the entire data distribution or at a local scale, in the vicinity of a given location in the distribution.

An important measure of global complexity is intrinsic dimensionality, which captures the effective number of degrees of freedom needed to describe the entire dataset. On the other hand, *local intrinsic dimensionality (LID)* [2] is capable of characterizing the complexity of the data distribution around a specified query location, thus capturing the number of degrees of freedom present at a local scale. LID is a unitless quantity that can also be interpreted as a relative growth rate of probability measure within an expanding neighborhood around the specified query location, or the intrinsic dimension of the space immediately around the query point.

Our focus in this paper is to characterize entropy and statistical divergences at a highly local scale, for an asymptotically small vicinity around a specified location. We show that it is possible to leverage properties that arise from LID based characterizations of lower tail distributions [3], to develop analytical expressions for a wide selection of entropy variants and statistical divergences, in both univariate and multivariate settings. This yields expressions for *tail entropies* and *tail divergences*.

Analytical characterizations for tail divergences and tail entropies are appealing from a number of perspectives. These are as follows:

- For univariate scenarios, if working with the tail of a distribution that has a single variable, we can conduct:
  – Temporal analysis: when a distribution models some property varying over time (e.g., survival analysis), we can analyze the entropy of a univariate distribution within an asymptotically short window of time, or the divergence between two univariate distributions within an asymptotically short window of time.
  – Distance-based analysis: when a distribution models distances from a query location to its nearest neighbors and the distances are induced by a global data distribution. Here, our results can be used for analysis of tail entropy or divergence between distributions within an asymptotically small distance interval. In the case of the latter, this can provide insight into multivariate properties, since under minimal assumptions the divergences between univariate distance distributions provide lower bounds for distances between multivariate distributions [4,5]. This is applicable for models such as generative adversarial networks (GANs), where it is important to test correspondence between synthetic and true distributions at a local level [6].
- For multivariate scenarios where we are analyzing distributions with multiple variables:
  – If an assumption of locally spherical symmetry of the distribution holds, then we can directly compute the tail entropy of a distribution or the divergence between two tail distributions in the vicinity of a single point. Such an assumption is suitable for analyzing data distributions for many types of physical systems such as fluids, glasses, metals and polymers, where local isotropy holds.

A key challenge in developing analytical characterizations for tail entropies and tail divergences is how to avoid or minimize assumptions about the form of the local distribution in the vicinity of the query (for example, assumptions such as a local normal distribution or a local uniform distribution). As we will see, analytical results are in fact possible—as the neighborhood radius asymptotically tends to zero, the tail distribution (a truncated distribution induced from the global distribution) is guaranteed to converge to a generalized pareto distribution (GPD), with the GPD parameter determined by the LID value of the tail distribution. The technical challenge is to rigorously delineate under what circumstances it is possible to leverage this relationship to achieve a dramatic simplification of the integrals that are required to compute varieties of tail entropy or distribution divergences. Our results in this paper show that such simplifications are in fact possible, for a wide range of tail entropies and divergences. This allows us to characterize and analyze fundamental properties of local neighborhood geometry, with results holding asymptotically for essentially all smooth data distributions.

In summary, our key contributions are the development of substantial new theory that asymptotically relates tail entropy, divergences and LID. It builds on and extends an earlier work by Bailey et al. [3], which focused solely on univariate entropies, without reference to divergences or multivariate settings. Specifically in this paper, we:

- Formulate technical lemmas which delineate when it is possible to substitute certain types of tail distributions by simple formulations that depend only on their associated LID values.
- Use these lemmas to compute univariate tail formulations of entropy, cross entropy, cumulative entropy, entropy power and generalized *q*-entropies, all in terms of the LID values of the original tail distributions.
- Use these lemmas to compute tail formulations of univariate statistical divergences and distances (Kullback–Leibler divergence, Jensen–Shannon divergence, Hellinger distance, $\chi^{2}$ divergence, α-divergence, Wasserstein distance and $L_{2}$ distance).
- Extend the univariate results to a multivariate context, when local spherical symmetry of the distribution holds.

## 2. Related Work

The core of our study involves intrinsic dimensionality (ID) and we begin by reviewing previous work on this topic.

There is a long history of work on ID, and this can be assessed either globally (for every data point) or locally (with respect to a chosen query point). Surveys of the field provide a good overview [7,8,9]. In the global case, a range of previous works have focused on topological models and appropriate estimation methods [10,11,12]. Such examples encompass techniques such as PCA and its variants [13], graph based methods [14] and fractal models [7,15]. Other approaches such as IDEA [16,17], DANCo [18] or 2-NN estimate the (global) intrinsic dimension based on concentration of norms and angles, or 2-nearest neighbors [19].

Local intrinsic dimensionality focuses on the intrinsic dimension of a particular query point and has been used in a range of applications. These include modeling deformation in granular materials [20,21], climate science [22,23], dimension reduction via local PCA [24], similarity search [25], clustering [26], outlier detection [27], statistical manifold learning [28], adversarial example detection [29], adversarial nearest neighbor characterization [30,31] and deep learning understanding [32,33]. In deep learning, it has been shown that adversarial examples are associated with high LID estimates, a characteristic that can be leveraged to build accurate adversarial example detectors [29]. It has also been found that the LID of deep representations [33] learned by Deep Neural Networks (DNNs) or the raw input data [34,35] is correlated with the generalization performance of DNNs. A ‘dimensionality expansion’ phenomenon has been observed when DNNs overfit to noisy class labels [32] and this can be leveraged to develop improved loss functions. The use of a “cross-LID” measure to evaluate the quality of synthetic examples generated by GANs has been proposed in [36]. Connections between local intrinsic dimensionality and global intrinsic dimensionality were explored by Romano et al in [37]. In the area of climate science and dynamical systems, a formulation similar to local intrinsic dimensionality has been developed and referred to as local dimension or instantaneous dimension [22,23,38], using links to extreme value theoretic methods. It has proved useful as measure to characterize predictability of states and explain system dynamics.

For local intrinsic dimensionality, a popular estimator is the maximum likelihood estimator, studied in the Euclidean setting by Levina and Bickel [39] and later formulated under the more general assumptions of extreme value theory by Houle [2] and Amsaleg et al. [40], who showed it to be equivalent to the classic Hill estimator [41]. Other local estimators include expected simplex skewness [42], the tight locality estimator [43], the MiND framework [17], manifold adaptive dimension [44], statistical distance [45] and angle-based approaches [46]. Smoothing approaches for estimation have also been used with success [47,48].

Local intrinsic dimensionality is closely related to (univariate) distance distributions. Fundamental relations for interpoint distances, connecting multivariate distributions and univariate distributions have been explored by both [4,5]. The former showed that two multivariate distributions are equal whenever the interpoint distances both within and between samples have the same univariate distribution, while the latter showed that two multivariate distributions *F* and *G* are different if their univariate distance distributions from some randomly chosen point *z* are different. This can form the basis of a two sample test for comparing *F* and *G*. These studies have implications for our work in this paper, since they characterize the role that comparison between univariate distributions can play as a necessary condition for comparing equality of multivariate distributions.

Our work in this paper formulates results for different varieties of entropy and different types of divergences. Entropy is a fundamental notion used across many scientific disciplines. A good overview of its role in information theory is presented in [49]. Entropy power (the exponential of entropy) is commonly used in signal processing and information theory, and is a building block for the well-known Shannon entropy power inequality which can be used to analyze the convolution of two independent random variables [50]. Entropy power goes under the name of perplexity in the field of natural language processing [51] and true diversity in the field of ecology [52]. It also corresponds to the volume of the smallest set that contains most of the probability measure [49], and it can be interpreted as a measure of statistical dispersion [53]. It is also related to Fisher information via Stam’s inequality [54].

Cumulative entropy was formulated in [55] and is a modification of cumulative residual entropy [56]. It is popular in reliability theory where it is used to characterize uncertainty over time intervals. Apart from reliability theory analysis, it has been used in data mining tasks such as dependency analysis [57] and subspace cluster analysis [58], where it has proved more effective due to good estimation properties. These data mining investigations have used cumulative entropy at a global level (over the entire data domain), rather than at the local (tail) level, as in our study. Generalized variants based on Tsallis *q*-statistics have been developed for both entropy [59] and cumulative entropy [60]. Inclusion of the extra *q* parameter can assist with higher robustness to anomalies and better fitting to characteristics of data distributions. Tail entropy has been used in financial applications for measuring the expected shortfall [61] in the upper tail using quantization. This is different from our context, where the our exclusive focus is on lower tails and we develop exact results for an asymptotic regime where lower tail size approaches zero.

Divergences between probability distributions are a fundamental building block in statistics and are used to assess the degree to which one probability distribution is different from another probability distribution. They have a wide range of formulations [1] and applications, which range from use as objective functions in supervised and unsupervised machine learning [62], to hypothesis and two sample or goodness of fit testing in statistics [63], as well as generative modeling in deep learning, particularly using the Wasserstein distance [64]. Asymptotic forms of KL divergence have been investigated by Contreras-Reyes [65], for comparison of multivariate asymmetric heavy-tailed distributions.

Finally, we note that this work considerably expands a recent study by Bailey et al. [3], which established relationships between tail entropies and LID. This current paper extends and generalizes that work in several directions: (i) We establish general lemmas that provide sufficient conditions for when it is possible to substitute a tail distribution with components such as a power law, inside an integral. The techniques of [3] were specially crafted for specific integrals. (ii) We provide results for statistical divergences and distances (the work of [3] only considers entropy). (iii) We show how to formulate results for the multivariate context (as [3] only considers univariate scenarios).

## 3. Local Intrinsic Dimensionality

In this section, we summarize the LID model using the presentation of [2]. LID can be regarded as a continuous extension of the expansion dimension [66,67]. Like earlier expansion-based models of intrinsic dimension, its motivation comes from the relationship between volume and radius in an expanding ball, where (as originally stated in [68]) the volume of the ball is taken to be the probability measure associated with the region it encloses. The probability as a function of radius—denoted by F(r)—has the form of a univariate cumulative distribution function (CDF). The model formulation (as stated in [2]) generalizes this notion to real-valued functions *F* for which F(0)=0, under appropriate assumptions of smoothness.

**Definition** **1** ([2])**.** *Let F be a real-valued function that is non-zero over some open interval containing* r∈R, r≠0. *The* intrinsic dimensionality of *F* at *r* *is defined as follows whenever the limit exists:*

$$\begin{array}{ccc} {\mathrm{IntrDim}}_{F}\left(r\right) & ≜ & \lim_{\epsilon \to 0}\frac{\ln\left(F((1+\epsilon )r)/F(r)\right)}{\ln(1+\epsilon )}\;. \end{array}$$

When *F* satisfies certain smoothness conditions in the vicinity of *r*, its intrinsic dimensionality has a convenient known form:

**Theorem** **1** ([2])**.** *Let F be a real-valued function that is non-zero over some open interval containing r∈R, r≠0. If F is continuously differentiable at r and using $F^{\prime }\left(r\right)$ to denote the derivative $\frac{dF(r)}{dr}$, then*

$${\mathrm{ID}}_{F}\left(r\right)\;≜\;\frac{r\cdot F^{\prime }\left(r\right)}{F(r)}\;=\;{\mathrm{IntrDim}}_{F}\left(r\right)\;.$$

Let x be a location of interest within a data domain S for which the distance measure $d:S\times S\to R_{+}\cup 0$ has been defined. To any generated sample s∈S we associate the distance d(x,s); in this way, a *global* distribution that produces the sample s can be said to induce the random value d(x,s) from a *local* distribution of distances taken with respect to x. The CDF F(r) of the local distance distribution is simply the probability of the sample distance lying within a threshold *r*—that is, F(r)≜Pr[d(x,s)≤r]. In characterizing the local intrinsic dimensionality in the vicinity of location x, we are interested in the limit of ${\mathrm{ID}}_{F}\left(r\right)$ as the distance *r* tends to 0, which we denote by

$${\mathrm{ID}}_{F}^{*}≜\lim_{r\to 0}{\mathrm{ID}}_{F}\left(r\right)\;.$$

Henceforth, when we refer to the local intrinsic dimensionality (LID) of a function *F*, or of a point x whose induced distance distribution has *F* as its CDF, we will take ‘LID’ to mean the quantity ${\mathrm{ID}}_{F}^{*}$. In general, ${\mathrm{ID}}_{F}^{*}$ is not necessarily an integer. In practice, estimation of the LID at x would give an indication of the dimension of the submanifold containing x that best fits the distribution.

The function ${\mathrm{ID}}_{F}$ can be seen to fully characterize its associated function *F*. This result is analogous to a foundational result from the statistical theory of extreme values (EVT), in that it corresponds under an inversion transformation to the Karamata representation theorem [69] for the upper tails of regularly varying functions. For more information on EVT and how the LID model relates to the extreme-value theoretic generalized pareto distribution, we refer the reader to [2,70,71].

**Theorem** **2 (LID Representation Theorem** **[2]).** *Let F:R→R be a real-valued function, and assume that ${\mathrm{ID}}_{F}^{*}$ exists. Let x and w be values for which x/w and F(x)/F(w) are both positive. If F is non-zero and continuously differentiable everywhere in the interval [min{x,w},max{x,w}], then*

$$\begin{array}{ccc} \frac{F(x)}{F(w)}\;=\;{\left(\frac{x}{w}\right)}^{{\mathrm{ID}}_{F}^{*}}\cdot A_{F}\left(x,w\right), & \;\mathrm{where} & \;A_{F}\left(x,w\right)\;≜\;\exp\left(\int_{x}^{w}\frac{{\mathrm{ID}}_{F}^{*}-{\mathrm{ID}}_{F}\left(t\right)}{t}\;dt\right), \end{array}$$

*whenever the integral exists.*

In [2], conditions on *x* and *w* are provided for which the factor $A_{F}\left(x,w\right)$ can be seen to tend to 1 as x,w→0. The convergence characteristics of *F* to its asymptotic form are expressed by the factor $A_{F}\left(x,w\right)$, which is related to the slowly varying component of functions as studied in EVT [70]. As we will shown in the next section, we make use of the LID Representation Theorem in our analysis of the limits of tail entropy variants under a form of normalization.

## 4. Definitions of Tail Entropies and Tail Dissimilarity Measures

In this section, we present the formulations of entropy, divergences and distances that will be studied in the later sections, in the light of the model of local intrinsic dimensionality outlined in Section 3. These entropies and dissimilarity measures will all be conditioned on the lower tails of smooth functions on domains bounded from below at zero. In each case, the formulations involve one or more non-negative real-valued functions whose restriction to [0,w] satisfies certain smooth growth properties:

**Definition** **2.** *Let*$F:R_{+}\cup 0\to R_{+}\cup 0$*be a function that is positive except at*F(0)=0. *We say that F is a* smooth growth function *if*

- *There exists a value r>0 such that F is monotonically increasing over (0,r);*
- *F is continuous over [0,r);*
- *F is differentiable over (0,r); and*
- *The local intrinsic dimensionality ${\mathrm{ID}}_{F}^{*}$ exists and is positive.*

Given a smooth growth function *F* and a value w>0, we define $F_{w}\left(t\right)≜\frac{F(t)}{F(w)}$. If *F* is the CDF of some random variable X≥0, then $F_{w}\left(t\right)=\mathrm{Pr}\left[X\leq t\;|X\leq w\right]$, which can in turn be interpreted as the CDF of the distribution of *X* conditioned to the lower tail [0,w]. It is easy to see that for a sufficiently small choice of *w*, $F_{w}$ must also be a smooth growth function. Its derivative $F_{w}^{\prime }\left(t\right)=\frac{F^{\prime }\left(t\right)}{F(w)}$ exists since $F^{\prime }\left(t\right)$ exists, and thus can be regarded as the probability density function (PDF) of the restriction of *F* to [0,w]. In addition, it can easily be shown (using Theorem 1) that the LID of $F_{w}$ is identical to that of *F*.

If the monotonicity of the function *F* is strict over the domain of interest [0,r), its inverse function $F^{-1}$ exists and satisfies the smooth growth conditions within some neighborhood of the origin. Moreover, $F_{w}^{-1}$ is also a smooth growth function over [0,1], with $F_{w}^{-1}\left(0\right)=0$ and $F_{w}^{-1}\left(1\right)=w$.

The following tail entropy, tail divergence and tail distance formulations all apply to any functions *F* and *G* satisfying the conditions stated above; in particular, they involve one or more of $F_{w}$, $F_{w}^{\prime }$, $G_{w}$, $G_{w}^{\prime }$, and (if the monotonicity of the functions is strict) $F_{w}^{-1}$ and $G_{w}^{-1}$. In their definitions, the only difference between the tail variants and the original versions is that the distributions are conditioned on the lower tail [0,w]. In the tail measures involving one or more of $F_{w}$, $F_{w}^{\prime }$, $G_{w}$ and $G_{w}^{\prime }$, integration is performed over the lower tail and not the entire distributional range [0,+∞); for the variant involving $F_{w}^{-1}$ and $G_{w}^{-1}$, integration is performed over [0,1] for values of *w* constrained to the lower tail.

We begin with (differential) tail entropy. Entropy is perhaps the most fundamental and widely used model of data complexity and can be regarded as a measure of the uncertainty of a distribution. Differential entropy assesses the expected surprisal of a random variable and can take negative values.

**Definition** **3** **(Tail Entropy).** *The entropy of F conditioned on [0,w] is*

$$H\left(F,w\right)≜-\int_{0}^{w}F_{w}^{\prime }\left(t\right)\ln F_{w}^{\prime }\left(t\right)\;dt\;.$$

The tail entropy is equal to $E(-\log F_{w}^{\prime })$, the expected value of the (tail) log-likelihood. It is also possible to define the variance of the (tail) log-likelihood. This is known as the *varentropy*. Understanding this further, note that one may define the information content of a random variable *X* with density function *f*, to be −logf(X). The entropy (uncertainty) then corresponds to the expected value of the information content of *X* and the varentropy corresponds to the variance of the information content of *X*. The varentropy was introduced by Song [72] as an intrinsic measure of the shape of a distribution and has been explored in a range of studies [73,74,75].

**Definition** **4** **(Tail varentropy).** *The varentropy of F conditioned on [0,w] is*

$$\mathrm{VarH}\left(F,w\right)≜\int_{0}^{w}F_{w}^{\prime }\left(t\right)\ln^{2}F_{w}^{\prime }\left(t\right)\;dt\;-{\left(\int_{0}^{w}F_{w}^{\prime }\left(t\right)\ln F_{w}^{\prime }\left(t\right)\;dt\right)}^{2}$$

The cumulative entropy is a variant of entropy proposed in [55,56] due to its attractive theoretical properties. Tail conditioning on the cumulative entropy has the same general form as that of the tail entropy. Cumulative entropy [55,56] is an information-theoretic measure popular in reliability theory, where it is used to model uncertainty over time intervals. It corresponds to the expected value of the mean inactivity time. Compared to ordinary Shannon differential entropy, cumulative entropy has certain attractive properties, such as non-negativity and ease of estimation.

**Definition** **5** **(Cumulative Tail Entropy).** *The cumulative entropy of F conditioned on [0,w] is*

$$\mathrm{cH}\left(F,w\right)≜-\int_{0}^{w}F_{w}\left(t\right)\ln F_{w}\left(t\right)\;dt\;.$$

The entropy power is the exponential of the entropy, and is also known as *perplexity* in the natural language processing community. It corresponds to the volume of the smallest set that contains most of the probability measure [49], and can be interpreted as a measure of statistical dispersion [53]. There are several standard definitions of entropy power in the research literature. For our purposes, we adopt the simplest—the exponential of Shannon entropy—for our definition conditioned to the tail.

**Definition** **6** **(Tail Entropy Power).** *The entropy power of F conditioned on [0,w] is defined to be*

HP(F,w)≜exp(H(F,w)).

In the introduction, we briefly mentioned some motivation for the entropy power HP(F,w). We can add to this as follows:

- It can be interpreted as a diversity. Observe that when *F* is a (univariate) uniform distance distribution ranging over the interval [0,w], we have ${\mathrm{ID}}_{F}^{*}=1$ and HP(F,w)=w. In other words, the entropy power is equal to the ‘effective diversity’ of the distribution (the number of neighbor distance possibilities).
- Given two different queries, each with its own neighborhood, one query with tail entropy power equal to 2 and the other with tail entropy power equal to 4, we can say that the distance distribution of the second query is twice as diverse as that of the first query.

For each of the tail entropy variants introduced above, we also propose analogous variants based on the *q*-entropy formulation due to Tsallis [59]. Generalized Tsallis entropies [59,60] are a family of entropies characterized via an exponent parameter *q* applied to the probabilities, in which the traditional (Shannon) entropy variants are obtained as the special case when *q* is allowed to tend to 1. The use of such a parameter *q* can often facilitate more accurate fitting of data characteristics and robustness to outliers.

**Definition** **7** **(Tail *q*-Entropy).** *For any q>0 (q≠1), the q-entropy of F conditioned on [0,w] is defined to be*

$$\begin{array}{ccc} H_{q}\left(F,w\right) & ≜ & \frac{1}{q-1}\left(1-\int_{0}^{w}{\left(F_{w}^{\prime }\left(t\right)\right)}^{q}\;dt\right)\;=\;\frac{1}{q-1}\int_{0}^{w}F_{w}^{\prime }\left(t\right)-{\left(F_{w}^{\prime }\left(t\right)\right)}^{q}\;dt\;. \end{array}$$

**Definition** **8** **(Cumulative Tail *q*-Entropy).** *For any q>0 (q≠1), the cumulative q-entropy of F conditioned on [0,w] is defined to be*

$$\begin{array}{c} {\mathrm{cH}}_{q}\left(F,w\right)≜\frac{1}{q-1}\int_{0}^{w}F_{w}\left(t\right)-{\left(F_{w}\left(t\right)\right)}^{q}\;dt\;. \end{array}$$

We define the tail *q*-entropy power using the *q*-exponential function from Tsallis statistics [59], $\exp_{q}\left(x\right)≜{\left[1+\left(1-q\right)x\right]}^{\frac{1}{1-q}}$. Note that L’Hôpital’s rule can be used to show that $\exp_{q}\left(x\right)\to e^{x}$ as q→1.

**Definition** **9** **(Tail *q*-Entropy Power).** *For any q>0 (q≠1), the q-entropy power of F conditioned on [0,w] is defined to be*

$${\mathrm{HP}}_{q}\left(F,w\right)\;\;≜\;\;{\left[1+\left(1-q\right)H_{q}\left(F,w\right)\right]}^{\frac{1}{1-q}}\;.$$

We next define the tail cross entropy. Cross entropy can be used to compare two probability distributions and is often employed as a loss function in machine learning, comparing a true distribution and a learned distribution. From an information theoretic perspective, cross entropy corresponds to the expected coding length when a wrong distribution *G* is assumed while the data actually follows a distribution *F*.

**Definition** **10** **(Tail Cross Entropy).** *The cross entropy from F to G, conditioned on [0,w], is defined to be*

$$\mathrm{XH}\left(F;G,w\right)\;\;≜\;\;{-\int }_{0}^{w}F_{w}^{\prime }\left(t\right)\ln G_{w}^{\prime }\left(t\right)\;dt\;.$$

Similar to entropy power, we can also define the cross entropy power, which is the exponential of the cross entropy.

**Definition** **11** **(Tail Cross Entropy Power).** *The cross entropy power from F to G, conditioned on [0,w], is defined to be*

$$\mathrm{XHP}\left(F;G,w\right)\;\;≜\;\;\exp\left({-\int }_{0}^{w}F_{w}^{\prime }\left(t\right)\ln G_{w}^{\prime }\left(t\right)\;dt\right)\;.$$

A classic and fundamental method for comparing two probability distributions is the Kullback–Leibler divergence (KL Divergence) [76]. KL(F,G) measures the degree to which a probability distribution *G* is different from a reference probability distribution *F*. It is a member of both the family of *f*-divergences and Bregman divergences. It is widely used in statistics, machine learning and information theory.

**Definition** **12** **(Tail KL Divergence).** *The Kullback–Leibler divergence from F to G, conditioned on [0,w], is defined to be*

$$\mathrm{KL}\left(F;G,w\right)\;\;≜\;\;\int_{0}^{w}F_{w}^{\prime }\left(t\right)\ln\frac{F_{w}^{\prime }\left(t\right)}{G_{w}^{\prime }\left(t\right)}\;dt\;.$$

The tail KL divergence can be connected to the tail entropy and the tail cross entropy according to the relationship KL(F;G,w)=XH(F;G;w)−H(F,w).

The Jensen–Shannon divergence (JS divergence) [77] is another popular measure of distance between probability distributions. It is based on the KL divergence, but unlike the KL, the square root of the JS divergence is a true metric.

**Definition** **13** **(Tail JS Divergence).** *The Jensen–Shannon divergence between F and G, conditioned on [0,w], is defined to be*

$$\mathrm{JS}\left(F;G,w\right)\;\;≜\;\;\frac{\mathrm{KL}(F;M,w)+\mathrm{KL}(G;M,w)}{2}\;,\;\;\mathrm{where}\;\;M\left(t\right)\;\;=\;\;\frac{F(t)+G(t)}{2}\;.$$

The tail JS divergence can also be written in terms of the tail entropies $\mathrm{JS}\left(F;G,w\right)=H\left(\frac{F+G}{2},w\right)-\frac{H(F,w)+H(G,w)}{2}$

The L2 distance is the squared Euclidean distance when comparing two probability distributions. It is part of the family of β divergences when setting β=2 [78].

**Definition** **14** **(Tail L2 Distance).** *The L2 distance between F and G, conditioned on [0,w], is defined to be*

$$L2D\left(F;G,w\right)\;\;≜\;\;\int_{0}^{w}{\left(F_{w}^{\prime }\left(t\right)-G_{w}^{\prime }\left(t\right)\right)}^{2}\;dt\;.$$

The Hellinger distance [79] is a true metric for comparing two probability distributions. The squared Hellinger distance a member of the family of *f*-divergences and is part of the family of α divergences when setting $\alpha =\frac{1}{2}$ [80].

**Definition** **15** **(Tail Hellinger Distance).** *The Hellinger distance between F and G, conditioned on [0,w], is defined to be*

$$\mathrm{HD}\left(F;G,w\right)\;\;≜\;\;\sqrt{\frac{1}{2}\int_{0}^{w}{\left(\sqrt{F_{w}^{\prime }\left(t\right)}-\sqrt{G_{w}^{\prime }\left(t\right)}\right)}^{2}\;dt}\;.$$

The $\chi^{2}$ divergence between two probability distributions [81] is a member of the family of *f* divergences and is part of the family of α divergences when setting α=2 [80].

**Definition** **16** **(Tail** $\chi^{2}$ **-Divergence).** *The $\chi^{2}$ divergence between F and G, conditioned on [0,w], is defined to be*

$$\chi^{2}D\left(F;G,w\right)\;\;≜\;\;\int_{0}^{w}\frac{{\left(F_{w}^{\prime }\left(t\right)-G_{w}^{\prime }\left(t\right)\right)}^{2}}{G_{w}^{\prime }\left(t\right)}\;dt\;.$$

The asymmetric α-divergence [80] is another member of the family of *f* divergences. When α=2 it is proportional to the $\chi^{2}$ divergence. When α=0.5 it is proportional to the squared Hellinger distance. When α→1 it corresponds to the KL-divergence.

**Definition** **17** **(Tail** α **-Divergence).** *The α-divergence from F to G, conditioned on [0,w], is defined to be*

$$\alpha D\left(F;G,w\right)\;\;≜\;\;\frac{1}{\alpha (1-\alpha )}\int_{0}^{w}\alpha F_{w}^{\prime }\left(t\right)+\left(1-\alpha \right)G_{w}^{\prime }\left(t\right)-{\left(F_{w}^{\prime }\left(t\right)\right)}^{\alpha }{\left(G_{w}^{\prime }\left(t\right)\right)}^{1-\alpha }\;dt\;.$$

The Wasserstein distance between two probability distributions is also known as the Kantorovich–Rubinstein metric [82] or the earth mover’s distance. It has become very popular as part of the loss function used in generative adversarial networks [83]. In the univariate case it can be expressed in a simple analytic form.

**Definition** **18** **(Tail Wasserstein Distance).** *The p-th Wasserstein distance between F and G, conditioned on [0,w], is defined to be*

$${\mathrm{WD}}_{p}\left(F;G,w\right)\;\;≜\;\;{\left(\int_{0}^{1}{\left|F_{w}^{-1}\left(u\right)-G_{w}^{-1}\left(u\right)\right|}^{p}\;du\right)}^{\frac{1}{p}}.$$

For some of the aforementioned tail measures, we will also consider a normalization of the entropy, divergence or distance (as the case may be) with respect to *w*, the length of the tail. In Section 5 and Section 6, we will show that as *w* tends to zero, the limits of these (possibly normalized) tail entropies and tail divergences can be expressed in terms of the local intrinsic dimensionalities of *F* and *G*. The notation for these variants, and our results for their limits in terms of ${\mathrm{ID}}_{F}^{*}$ and ${\mathrm{ID}}_{G}^{*}$, are summarized in Table 1.

## 5. Simplification of Tail Measures

Next, we present the main theoretical contributions of the paper: three technical lemmas that will later be used to establish relationships between local intrinsic dimensionality and a variety of tail measures based on entropy, divergences or distances. The results presented in this section all apply *asymptotically*, as the tail boundary tends toward zero.

Each of the three lemmas allow, under certain conditions, the simplification of limits of integrals involving smooth growth functions of the form $F_{w}$ (as defined in Section 4), or its associated first derivative $F_{w}^{\prime }$ or inverse function $F_{w}^{-1}$. The limit integral simplifications allow for the substitution of the function (or derivative or inverse) by expressions that involve one or more of the following: the LID value of the function, the variable of integration or the tail boundary *w*. Moreover, the lemmas require that the integrand be monotone with respect to small variations in the targeted function.

The first lemma allows terms of the form $F_{w}$ (resembling the CDF of a tail-conditioned distribution) to be converted into a term that depends only on the variable of integration, the tail length *w*, and the local intrinsic dimension ${\mathrm{ID}}_{F}^{*}$.

**Lemma** **1.** *Let F be a smooth growth function over the interval [0,r). Consider the function $\phi :R_{+}^{2}\to R$ admitting a representation of the form*

$$\phi \left(t,w\right)\;\equiv \;\psi \left(t,w,z(t,w)\right),$$

*where:*

- *$\psi :R_{+}^{3}\to R$;*
- *$z\left(t,w\right)=F_{w}\left(t\right)=\frac{F(t)}{F(w)}$; and*
- *for all fixed choices of t and w satisfying 0<t≤w<r, ψ(t,w,z) is monotone and continuously partially differentiable with respect to z over the interval z∈(0,1].*

*Then*

$$\begin{array}{ccc} \lim_{w\to 0^{+}}\int_{0}^{w}\phi \left(t,w\right)\;dt\; & \equiv & \lim_{w\to 0^{+}}\int_{0}^{w}\psi \left(t,w,F_{w}\left(t\right)\right)\;dt\\ & = & \lim_{w\to 0^{+}}\int_{0}^{w}\psi \left(t,w,{\left(\frac{t}{w}\right)}^{{\mathrm{ID}}_{F}^{*}}\right)\;dt \end{array}$$

*whenever the latter limit exists or diverges to +∞ or −∞.*

**Proof.**  Since *F* is assumed to be a smooth growth function, the limit ${\mathrm{ID}}_{F}^{*}=\lim_{v\to 0^{+}}{\mathrm{ID}}_{F}\left(v\right)$ exists and is positive. We present an ‘epsilon-delta’ argument based on this limit. For any real value ϵ>0 satisfying $\epsilon <\min\{r,{\mathrm{ID}}_{F}^{*}\}$, there must exist a value 0<δ<ϵ such that v<δ implies that $|{\mathrm{ID}}_{F}\left(v\right)-{\mathrm{ID}}_{F}^{*}|<\epsilon$. Therefore, when 0<t≤w<δ,

$$\begin{array}{ccc} \left|\ln A_{F}\left(t,w\right)\right| & \;=\; & \left|\int_{t}^{w}\frac{{\mathrm{ID}}_{F}^{*}-{\mathrm{ID}}_{F}\left(v\right)}{v}\;dv\right|\;<\;\epsilon \cdot \left|\int_{t}^{w}\frac{1}{v}\;dv\right|\;\;=\;\;\epsilon \cdot \ln\frac{w}{t}\;. \end{array}$$

Exponentiating, we obtain the bounds

$${\left(\frac{w}{t}\right)}^{-\epsilon }\;\;<\;\;A_{F}\left(t,w\right)\;\;<\;\;{\left(\frac{w}{t}\right)}^{\epsilon }\;.$$

Applying this bound together with Theorem 2, the ratio $F_{w}\left(t\right)=\frac{F(t)}{F(w)}$ can be seen to satisfy

(1) $${\left(\frac{t}{w}\right)}^{{\mathrm{ID}}_{F}^{*}+\epsilon }<\;\;\frac{F(t)}{F(w)}\;\;=\;\;A_{F}\left(t,w\right)\cdot {\left(\frac{t}{w}\right)}^{{\mathrm{ID}}_{F}^{*}}<\;\;{\left(\frac{t}{w}\right)}^{{\mathrm{ID}}_{F}^{*}-\epsilon }\;.$$

Over the domain of interest 0<t≤w<δ, the assumption that $0<\epsilon <\min\{r,{\mathrm{ID}}_{F}^{*}\}$ ensures that $0<\frac{t}{w}\leq 1$, and that the upper and lower bounds of Inequality (1) lie in the interval (0,1]. Since ψ(t,w,z) has been assumed to be monotone with respect to z∈(0,1], the maximum and minimum attained by ψ over choices of *z* restricted to any (closed) subinterval of (0,1] must occur at opposite endpoints of the subinterval. With this in mind, for any choice of $\epsilon \in (0,\min\left\{r,{\mathrm{ID}}_{F}^{*}\right\})$, Inequality (1) implies that

$$\begin{array}{ccc} B_{\min}\left(t,w,\epsilon \right)\;\;\leq & \psi \left(t,w,F_{w}\left(t\right)\right) & \leq \;\;B_{\max}\left(t,w,\epsilon \right)\\ \mathrm{and}\;\;\;\;\;\int_{0}^{w}B_{\min}\left(t,w,\epsilon \right)\;dt\;\;\leq & \int_{0}^{w}\psi \left(t,w,F_{w}\left(t\right)\right)\;dt & \leq \;\;\int_{0}^{w}B_{\max}\left(t,w,\epsilon \right)\;dt\;, \end{array}$$

where

$$\begin{array}{ccc} B_{\min}\left(t,w,\epsilon \right) & ≜ & \min\left\{\psi \left(t,w,{\left(\frac{t}{w}\right)}^{{\mathrm{ID}}_{F}^{*}-\epsilon }\right),\psi \left(t,w,{\left(\frac{t}{w}\right)}^{{\mathrm{ID}}_{F}^{*}+\epsilon }\right)\right\}\;,\\ B_{\max}\left(t,w,\epsilon \right) & ≜ & \max\left\{\psi \left(t,w,{\left(\frac{t}{w}\right)}^{{\mathrm{ID}}_{F}^{*}-\epsilon }\right),\psi \left(t,w,{\left(\frac{t}{w}\right)}^{{\mathrm{ID}}_{F}^{*}+\epsilon }\right)\right\}\;. \end{array}$$

Since ψ(t,w,z) and $\int_{0}^{w}\psi \left(t,w,z\right)\;dt$ are also continuously partially differentiable with respect to *z* over z∈(0,1],

$$\begin{array}{ccc} \lim_{\epsilon \to 0^{+}}B_{\min}\left(t,w,\epsilon \right)\;\;= & \lim_{\epsilon \to 0^{+}}B_{\max}\left(t,w,\epsilon \right) & =\;\;\psi \left(t,w,{\left(\frac{t}{w}\right)}^{{\mathrm{ID}}_{F}^{*}}\right)\;\;\;\;\;\mathrm{and}\\ \lim_{\overset{\epsilon \to 0^{+}}{w<\epsilon }}\int_{0}^{w}B_{\min}\left(t,w,\epsilon \right)\;dt\;\;= & \lim_{\overset{\epsilon \to 0^{+}}{w<\epsilon }}\int_{0}^{w}B_{\max}\left(t,w,\epsilon \right)\;dt & =\;\;\lim_{w\to 0^{+}}\int_{0}^{w}\psi \left(t,w,{\left(\frac{t}{w}\right)}^{{\mathrm{ID}}_{F}^{*}}\right)\;dt. \end{array}$$

It therefore follows from the squeeze theorem for integrals that

$$\begin{array}{ccc} \lim_{w\to 0^{+}}\int_{0}^{w}\psi \left(t,w,F_{w}\left(t\right)\right)\;dt & \;=\; & \lim_{w\to 0^{+}}\int_{0}^{w}\psi \left(t,w,{\left(\frac{t}{w}\right)}^{{\mathrm{ID}}_{F}^{*}}\right)\;dt\;, \end{array}$$

whenever the right-hand limit exists or diverges. □

In a manner similar to that of the preceding lemma, the following result allows terms of the form $F_{w}^{-1}$ (the inverse of $F_{w}$) to be converted into a term that depends only on the variable of integration, the tail length *w* and the local intrinsic dimension ${\mathrm{ID}}_{F}^{*}$. Here, in order to ensure the existence of the inverse function, *F* (and by extension $F_{w}$ and $F_{w}^{-1}$) must be strictly monotonically increasing over the tail.

**Lemma** **2.** *Let F be a smooth growth function over the interval [0,r). Let us also assume that, over the interval, the monotonicity of F is strict. Consider the function $\phi :R_{+}^{2}\to R$ admitting a representation of the form*

$$\phi \left(u,w\right)\;\equiv \;\psi \left(u,w,z(u,w)\right),$$

*where:*

- *$\psi :R_{+}^{3}\to R$;*
- *$z\left(u,w\right)=F_{w}^{-1}\left(u\right)$ for all w∈(0,r), where $F_{w}\left(t\right)≜F\left(t\right)/F\left(w\right)$ is restricted to values of t in [0,w]; and*
- *for all fixed choices of u and w satisfying u∈[0,1] and 0<w<r, ψ(u,w,z) is monotone and continuously partially differentiable with respect to z over the interval z∈(0,r).*

*Then*

$$\begin{array}{ccc} \lim_{w\to 0^{+}}\int_{0}^{1}\phi \left(u,w\right)\;du\; & \equiv & \lim_{w\to 0^{+}}\int_{0}^{1}\psi \left(u,w,F_{w}^{-1}\left(u\right)\right)\;du\\ & = & \lim_{w\to 0^{+}}\int_{0}^{1}\psi \left(u,w,wu^{\frac{1}{{\mathrm{ID}}_{F}^{*}}}\right)\;du \end{array}$$

*whenever the latter limit exists or diverges to +∞ or −∞.*

**Proof.**  First, we note that the strict monotonicity of *F* implies that for all u∈[0,1] and w∈(0,r), the function $F_{w}^{-1}\left(u\right)$ is uniquely defined when $F_{w}$ is restricted to [0,w].

As in the proof of Lemma 1, an ‘epsilon-delta’ argument based on the existence of the limit ${\mathrm{ID}}_{F}^{*}=\lim_{v\to 0^{+}}{\mathrm{ID}}_{F}\left(v\right)$ yields the following: for any real value ϵ>0 satisfying $\epsilon <\min\{r,{\mathrm{ID}}_{F}^{*}\}$, there exists a value δ∈(0,ϵ) such that

$${\left(\frac{t}{w}\right)}^{{\mathrm{ID}}_{F}^{*}+\epsilon }<\;\;F_{w}\left(t\right)\;\;=\;\;\frac{F(t)}{F(w)}\;\;<\;\;{\left(\frac{t}{w}\right)}^{{\mathrm{ID}}_{F}^{*}-\epsilon }$$

holds for all 0<t≤w<δ. Solving for *t* through exponentiation of the bounds, and then setting $t=F_{w}^{-1}\left(u\right)$, we obtain

$$\begin{array}{ccc} w\cdot {\left(F_{w}\left(t\right)\right)}^{\frac{1}{{\mathrm{ID}}_{F}^{*}-\epsilon }}\;\;< & t & <\;\;w\cdot {\left(F_{w}\left(t\right)\right)}^{\frac{1}{{\mathrm{ID}}_{F}^{*}+\epsilon }}\\ w\cdot {\left(F_{w}\left(F_{w}^{-1}\left(u\right)\right)\right)}^{\frac{1}{{\mathrm{ID}}_{F}^{*}-\epsilon }}\;\;< & \;F_{w}^{-1}\left(u\right)\; & <\;\;w\cdot {\left(F_{w}\left(F_{w}^{-1}\left(u\right)\right)\right)}^{\frac{1}{{\mathrm{ID}}_{F}^{*}+\epsilon }}\\ wu^{\frac{1}{{\mathrm{ID}}_{F}^{*}-\epsilon }}\;\;< & \;F_{w}^{-1}\left(u\right)\; & <\;\;wu^{\frac{1}{{\mathrm{ID}}_{F}^{*}+\epsilon }}\;. \end{array}$$

The remainder of the proof follows essentially the same path as that of Lemma 1. Over the domain of interest 0<t≤w<δ, the assumption that $0<\epsilon <\min\{r,{\mathrm{ID}}_{F}^{*}\}$ ensures that $0<\frac{t}{w}\leq 1$, and that *u* lies in the interval (0,w]. Since ψ(u,w,z) has been assumed to be monotone with respect to z∈(0,r), the maximum and minimum attained by ψ over choices of *z* restricted to any (closed) subinterval of (0,r) must occur at opposite endpoints. Therefore, for any choice of $\epsilon \in (0,\min\left\{r,{\mathrm{ID}}_{F}^{*}\right\})$,

$$\begin{array}{ccc} C_{\min}\left(u,w,\epsilon \right)\;\;\leq & \psi \left(u,w,F_{w}^{-1}\left(u\right)\right) & \leq \;\;C_{\max}\left(u,w,\epsilon \right)\\ \mathrm{and}\;\;\;\;\;\int_{0}^{1}C_{\min}\left(u,w,\epsilon \right)\;du\;\;\leq & \int_{0}^{1}\psi \left(u,w,F_{w}^{-1}\left(u\right)\right)\;du & \leq \;\;\int_{0}^{1}C_{\max}\left(u,w,\epsilon \right)\;du\;, \end{array}$$

where

$$\begin{array}{ccc} C_{\min}\left(u,w,\epsilon \right) & ≜ & \min\left\{\psi \left(u,w,wu^{\frac{1}{{\mathrm{ID}}_{F}^{*}-\epsilon }}\right),\psi \left(u,w,wu^{\frac{1}{{\mathrm{ID}}_{F}^{*}+\epsilon }}\right)\right\}\;,\\ C_{\max}\left(u,w,\epsilon \right) & ≜ & \max\left\{\psi \left(u,w,wu^{\frac{1}{{\mathrm{ID}}_{F}^{*}-\epsilon }}\right),\psi \left(u,w,wu^{\frac{1}{{\mathrm{ID}}_{F}^{*}+\epsilon }}\right)\right\}\;. \end{array}$$

Since ψ(u,w,z) is also continuously partially differentiable with respect to *z* over z∈(0,r),

$$\begin{array}{ccc} \lim_{\epsilon \to 0^{+}}C_{\min}\left(u,w,\epsilon \right)\;\;= & \lim_{\epsilon \to 0^{+}}C_{\max}\left(u,w,\epsilon \right) & =\;\;\psi \left(u,w,wu^{\frac{1}{{\mathrm{ID}}_{F}^{*}}}\right)\;\;\;\;\;\mathrm{and}\\ \lim_{\overset{\epsilon \to 0^{+}}{w<\epsilon }}\int_{0}^{1}C_{\min}\left(u,w,\epsilon \right)\;du\;\;= & \lim_{\overset{\epsilon \to 0^{+}}{w<\epsilon }}\int_{0}^{1}C_{\max}\left(u,w,\epsilon \right)\;du & =\;\;\lim_{w\to 0^{+}}\int_{0}^{1}\psi \left(u,w,wu^{\frac{1}{{\mathrm{ID}}_{F}^{*}}}\right)\;du. \end{array}$$

It therefore follows from the squeeze theorem for integrals that

$$\begin{array}{ccc} \lim_{w\to 0^{+}}\int_{0}^{1}\psi \left(u,w,F_{w}^{-1}\left(u\right)\right)\;du & \;=\; & \lim_{w\to 0^{+}}\int_{0}^{1}\psi \left(u,w,wu^{\frac{1}{{\mathrm{ID}}_{F}^{*}}}\right)\;du\;, \end{array}$$

whenever the right-hand limit exists or diverges. □

The third lemma facilitates the conversion of a term of the form $F_{w}^{\prime }$ to $F_{w}$, together with a factor that depends only on the variable of integration and ${\mathrm{ID}}_{F}^{*}$. Since *F* is assumed to be a smooth growth function, $F_{w}$ must be smooth as well, and therefore $F_{w}$ satisfies the conditions of Theorem 1 over [0,w). Hence, $F_{w}^{\prime }$ can be substituted by an expression involving $F_{w}$:

$$F_{w}^{\prime }\left(t\right)=\frac{{\mathrm{ID}}_{F_{w}}\left(t\right)}{t}\cdot F_{w}\left(t\right)=\frac{{\mathrm{ID}}_{F}\left(t\right)}{t}\cdot F_{w}\left(t\right)\;.$$

The substitution comes at the cost of introducing a non-constant factor ${\mathrm{ID}}_{F}\left(t\right)$. The following lemma shows that ${\mathrm{ID}}_{F}\left(t\right)$ can in turn be substituted by the constant ${\mathrm{ID}}_{F}^{*}$, provided that certain monotonicity assumptions are satisfied.

**Lemma** **3.** *Let F be a smooth growth function over the interval [0,r). Consider the function $\phi :R_{+}^{2}\to R$ admitting a representation of the form*

$$\phi \left(t,w\right)\;\equiv \;\psi \left(t,w,z(t,w)\right),$$

*where:*

- *$\psi :R_{+}^{3}\to R$;*
- *$z\left(t,w\right)={\mathrm{ID}}_{F}\left(t\right)$, and*
- *there exists a value $\gamma \in (0,{\mathrm{ID}}_{F}^{*})$ such that for all fixed choices of t satisfying 0<t≤w<r, ψ(t,w,z) is monotone with respect to z over the interval $z\in ({\mathrm{ID}}_{F}^{*}-\gamma ,{\mathrm{ID}}_{F}^{*}+\gamma )$.*

*Then*

$$\begin{array}{ccc} \lim_{w\to 0^{+}}\int_{0}^{w}\phi \left(t,w\right)\;dt\; & \equiv & \lim_{w\to 0^{+}}\int_{0}^{w}\psi \left(t,w,{\mathrm{ID}}_{F}\left(t\right)\right)\;dt\;=\;\lim_{w\to 0^{+}}\int_{0}^{w}\psi \left(t,w,{\mathrm{ID}}_{F}^{*}\right)\;dt \end{array}$$

*whenever the latter limit exists or diverges to +∞ or −∞.*

**Proof.**  Since *F* is assumed to be a smooth growth function, the limit ${\mathrm{ID}}_{F}^{*}=\lim_{v\to 0^{+}}{\mathrm{ID}}_{F}\left(v\right)$ exists and is positive. We present an ‘epsilon-delta’ argument based on this limit. For any real value ϵ>0 satisfying ϵ<min{r,γ}, there must exist a value 0<δ<ϵ such that v<δ implies that $|{\mathrm{ID}}_{F}\left(v\right)-{\mathrm{ID}}_{F}^{*}|<\epsilon$.

Since ψ(t,w,z) has been assumed to be monotone with respect to *z* over the interval $z\in ({\mathrm{ID}}_{F}^{*}-\gamma ,{\mathrm{ID}}_{F}^{*}+\gamma )$, the restriction v<δ<ϵ<min{r,γ} ensures that ψ(t,w,z) is monotone over the entire domain of interest 0<t≤w<δ. Therefore, the maximum and minimum attained by ψ over choices of *z* restricted to any (closed) subinterval of $\left({\mathrm{ID}}_{F}^{*}-\gamma ,{\mathrm{ID}}_{F}^{*}+\gamma \right)$ must occur at opposite endpoints of the subinterval. As in the proof of Lemma 1,

$$\begin{array}{ccc} D_{\min}\left(t,w,\epsilon \right)\;\;\leq & \psi \left(t,w,{\mathrm{ID}}_{F}\left(t\right)\right) & \leq \;\;D_{\max}\left(t,w,\epsilon \right)\\ \mathrm{and}\;\;\;\;\;\int_{0}^{w}D_{\min}\left(t,w,\epsilon \right)\;dt\;\;\leq & \int_{0}^{w}\psi \left(t,w,{\mathrm{ID}}_{F}\left(t\right)\right)\;dt & \leq \;\;\int_{0}^{w}D_{\max}\left(t,w,\epsilon \right)\;dt\;, \end{array}$$

where

$$\begin{array}{ccc} D_{\min}\left(t,w,\epsilon \right) & ≜ & \min\left\{\psi \left(t,w,{\mathrm{ID}}_{F}^{*}-\epsilon \right),\psi \left(t,w,{\mathrm{ID}}_{F}^{*}+\epsilon \right)\right\}\;,\\ D_{\max}\left(t,w,\epsilon \right) & ≜ & \max\left\{\psi \left(t,w,{\mathrm{ID}}_{F}^{*}-\epsilon \right),\psi \left(t,w,{\mathrm{ID}}_{F}^{*}+\epsilon \right)\right\}\;. \end{array}$$

Since ψ(t,w,z) is also continuously partially differentiable with respect to *z* over the range $\left({\mathrm{ID}}_{F}^{*}-\gamma ,{\mathrm{ID}}_{F}^{*}+\gamma \right)$,

$$\begin{array}{ccc} \lim_{\epsilon \to 0^{+}}D_{\min}\left(t,w,\epsilon \right)\;\;= & \lim_{\epsilon \to 0^{+}}D_{\max}\left(t,w,\epsilon \right) & =\;\;\psi \left(t,w,{\mathrm{ID}}_{F}^{*}\right)\;\;\;\;\;\mathrm{and}\\ \lim_{\overset{\epsilon \to 0^{+}}{w<\epsilon }}\int_{0}^{w}D_{\min}\left(t,w,\epsilon \right)\;dt\;\;= & \lim_{\overset{\epsilon \to 0^{+}}{w<\epsilon }}\int_{0}^{w}D_{\max}\left(t,w,\epsilon \right)\;dt & =\;\;\lim_{w\to 0^{+}}\int_{0}^{w}\psi \left(t,w,{\mathrm{ID}}_{F}^{*}\right)\;dt. \end{array}$$

It therefore follows from the squeeze theorem for integrals that

$$\begin{array}{c} \lim_{w\to 0^{+}}\int_{0}^{w}\psi \left(t,w,{\mathrm{ID}}_{F}\left(t\right)\right)\;dt\;\;=\;\lim_{w\to 0^{+}}\int_{0}^{w}\psi \left(t,w,{\mathrm{ID}}_{F}^{*}\right)\;dt\;, \end{array}$$

whenever the right-hand limit exists or diverges. □

## 6. Derivation of the Limits of Tail Measures

In this section, we will see how the three substitution lemmas can be applied to the limits of tail measures of entropy, divergence or distance, so as to produce formulations that depend only on the local intrinsic dimensions of the functions involved. All three lemmas require that the integral function be monotone with respect to small variations in the term that is targeted for substitution. In the discussion, we choose two tail measures as running examples: the tail KL divergence and the second tail Wasserstein distance (p=2).

### 6.1. Handling Derivatives of Smooth Growth Functions

In the case of the tail KL divergence, Theorem 1 allows us to substitute out the first derivatives $F_{w}^{\prime }$ and $G_{w}^{\prime }$ for the functions $F_{w}$ and $G_{w}$:

$$\mathrm{KL}\left(F;G,w\right)\;=\;\int_{0}^{w}F_{w}^{\prime }\left(t\right)\ln\frac{F_{w}^{\prime }\left(t\right)}{G_{w}^{\prime }\left(t\right)}\;dt\;=\;\int_{0}^{w}\frac{{\mathrm{ID}}_{F}\left(t\right)\;F_{w}\left(t\right)}{t}\ln\frac{{\mathrm{ID}}_{F}\left(t\right)\;F_{w}\left(t\right)}{{\mathrm{ID}}_{G}\left(t\right)\;G_{w}\left(t\right)}\;dt\;.$$

### 6.2. Substitution of LID Functions by Constants

In the limit of the tail KL divergence, the functions ${\mathrm{ID}}_{F}\left(t\right)$ and ${\mathrm{ID}}_{G}\left(t\right)$ can be replaced by the constants ${\mathrm{ID}}_{F}^{*}$ and ${\mathrm{ID}}_{G}^{*}$, respectively, through three successive applications of Lemma 3. To verify that the monotonicity condition of the Lemma is satisfied, we choose one of the terms and replace it by a new variable, *z*:

$$\lim_{w\to 0^{+}}\mathrm{KL}\left(F;G,w\right)\;=\;\lim_{w\to 0^{+}}\int_{0}^{w}\frac{z\;F_{w}\left(t\right)}{t}\ln\frac{{\mathrm{ID}}_{F}\left(t\right)\;F_{w}\left(t\right)}{{\mathrm{ID}}_{G}\left(t\right)\;G_{w}\left(t\right)}\;dt\;.$$

For any fixed values of *t* and *w*, it is easy to see that the integrand is locally monotone in the vicinity of $z={\mathrm{ID}}_{F}\left(t\right)$—here, if $\ln\frac{{\mathrm{ID}}_{F}\left(t\right)\;F_{w}\left(t\right)}{{\mathrm{ID}}_{G}\left(t\right)\;G_{w}\left(t\right)}$ is positive, a small increase in *z* (above the value ${\mathrm{ID}}_{F}\left(t\right)$) would result in an increase in the value of the integrand, and a small decrease would cause the integrand to decrease. If instead the logarithmic factor were negative, an increase in *z* would result in a decrease in the value of the integrand. Either way, the integrand would be monotone in the vicinity of $z={\mathrm{ID}}_{F}\left(t\right)$ at each fixed value of *t* and *w*. Its monotonicity condition thus being satisfied, Lemma 3 allows the targeted instance of ${\mathrm{ID}}_{F}\left(t\right)$ to be substituted by ${\mathrm{ID}}_{F}^{*}$:

$$\lim_{w\to 0^{+}}\mathrm{KL}\left(F;G,w\right)\;=\;\lim_{w\to 0^{+}}\int_{0}^{w}\frac{{\mathrm{ID}}_{F}^{*}\;F_{w}\left(t\right)}{t}\ln\frac{{\mathrm{ID}}_{F}\left(t\right)\;F_{w}\left(t\right)}{{\mathrm{ID}}_{G}\left(t\right)\;G_{w}\left(t\right)}\;dt\;.$$

Similarly, it can be verified that the new integrand is monotone in each of the remaining two factors ${\mathrm{ID}}_{F}\left(t\right)$ and ${\mathrm{ID}}_{G}\left(t\right)$; consequently, they too can be substituted by ${\mathrm{ID}}_{F}^{*}$ and ${\mathrm{ID}}_{G}^{*}$, one at a time, to yield

$$\lim_{w\to 0^{+}}\mathrm{KL}\left(F;G,w\right)\;=\;\lim_{w\to 0^{+}}\int_{0}^{w}\frac{{\mathrm{ID}}_{F}^{*}\;F_{w}\left(t\right)}{t}\ln\frac{{\mathrm{ID}}_{F}^{*}\;F_{w}\left(t\right)}{{\mathrm{ID}}_{G}^{*}\;G_{w}\left(t\right)}\;dt\;.$$

### 6.3. Elimination of Tail-Conditioned Smooth Growth Functions

Now that the tail KL divergence has been reformulated in terms of the tail-conditioned smooth growth functions $F_{w}$ and $G_{w}$, these two functions can be substituted out via three successive applications of Lemma 1, so as to obtain the limit of an integral involving only the variable of integration *t*, and the constants *w*, ${\mathrm{ID}}_{F}$ and ${\mathrm{ID}}_{G}$:

$$\lim_{w\to 0^{+}}\mathrm{KL}\left(F;G,w\right)\;=\;\lim_{w\to 0^{+}}\int_{0}^{w}\frac{{\mathrm{ID}}_{F}^{*}}{t}{\left(\frac{t}{w}\right)}^{{\mathrm{ID}}_{F}^{*}}\ln\left[\frac{{\mathrm{ID}}_{F}^{*}}{{\mathrm{ID}}_{G}^{*}}{\left(\frac{t}{w}\right)}^{{\mathrm{ID}}_{F}^{*}-{\mathrm{ID}}_{G}^{*}}\right]\;dt\;.$$

As in the previous step in which ${\mathrm{ID}}_{F}\left(t\right)$ and ${\mathrm{ID}}_{G}\left(t\right)$ were substituted out, the monotonicity conditions of Lemma 1 can easily be verified.

Now that the integral involves only constants and the variable *t*, it can be solved straightforwardly using the integration-by-parts technique, yielding

$$\lim_{w\to 0^{+}}\mathrm{KL}\left(F;G,w\right)\;=\;\lim_{w\to 0^{+}}\left(\frac{{\mathrm{ID}}_{G}^{*}}{{\mathrm{ID}}_{F}^{*}}-\ln\frac{{\mathrm{ID}}_{G}^{*}}{{\mathrm{ID}}_{F}^{*}}-1\right)\;=\;\frac{{\mathrm{ID}}_{G}^{*}}{{\mathrm{ID}}_{F}^{*}}-\ln\frac{{\mathrm{ID}}_{G}^{*}}{{\mathrm{ID}}_{F}^{*}}-1\;.$$

### 6.4. Elimination of the Inverses of Tail-Conditioned Smooth Growth Functions

We now turn our attention to the limit of the tail Wasserstein distance for the case p=2. Using Lemma 2, the inverse functions $F_{w}^{-1}$ and $G_{w}^{-1}$ can be substituted out, provided that the monotonicity requirements are satisfied. However, immediate application of the lemma to $F_{w}^{-1}\left(u\right)$ or $G_{w}^{-1}\left(u\right)$ does not necessarily work—to see this, consider substituting $F_{w}^{-1}\left(u\right)$ by the new variable *z*.

$${\mathrm{WD}}_{2}\left(F;G,w\right)\;=\;\sqrt{\int_{0}^{1}{\left(F_{w}^{-1}\left(u\right)-G_{w}^{-1}\left(u\right)\right)}^{2}\;du}\;=\;\sqrt{\int_{0}^{1}{\left(z-G_{w}^{-1}\left(u\right)\right)}^{2}\;du}\;.$$

Clearly, the integrand is not necessarily monotone in *z* in the vicinity of those values of the integration variable *u* where $G_{w}^{-1}\left(u\right)=z$.

Instead, we expand the squared difference and apply Lemma 3 to each of the resulting four occurrences of $F_{w}^{-1}$ and $G_{w}^{-1}$, one by one. By way of illustration, we consider substitution by *z* for the factor of $F_{w}^{-1}\left(u\right)$ in the cross term:

$$\begin{array}{ccc} \lim_{w\to 0^{+}}{\left({\mathrm{WD}}_{2}\left(F;G,w\right)\right)}^{2} & = & \lim_{w\to 0^{+}}\int_{0}^{1}{\left(F_{w}^{-1}\left(u\right)-G_{w}^{-1}\left(u\right)\right)}^{2}\;du\\ & = & \lim_{w\to 0^{+}}\int_{0}^{1}{\left(F_{w}^{-1}\left(u\right)\right)}^{2}-2F_{w}^{-1}\left(u\right)G_{w}^{-1}\left(u\right)+{\left(G_{w}^{-1}\left(u\right)\right)}^{2}\;du\\ & = & \lim_{w\to 0^{+}}\int_{0}^{1}{\left(F_{w}^{-1}\left(u\right)\right)}^{2}-2z\cdot G_{w}^{-1}\left(u\right)+{\left(G_{w}^{-1}\left(u\right)\right)}^{2}\;du\;. \end{array}$$

With respect to small variations in the variable *z* about the value $F_{w}^{-1}\left(u\right)$, noting that $G_{w}^{-1}$ is always non-negative, the integrand is easily seen to be monotone in *z* when $G_{w}^{-1}\left(u\right)$ is non-zero: for any increase in *z*, the value of the integrand decreases, and for any decrease in *z*, the value of the integrand increases. Lemma 2 can therefore be applied, producing

$$\begin{array}{ccc} \lim_{w\to 0^{+}}{\left({\mathrm{WD}}_{2}\left(F;G,w\right)\right)}^{2} & = & \lim_{w\to 0^{+}}\int_{0}^{1}{\left(F_{w}^{-1}\left(u\right)\right)}^{2}-2wu^{\frac{1}{{\mathrm{ID}}_{F}^{*}}}\cdot G_{w}^{-1}\left(u\right)+{\left(G_{w}^{-1}\left(u\right)\right)}^{2}\;du\;. \end{array}$$

After three more applications of Lemma 2, followed by taking the square root of the integral, we obtain

$$\begin{array}{ccc} \lim_{w\to 0^{+}}{\mathrm{WD}}_{2}\left(F;G,w\right) & = & \lim_{w\to 0^{+}}{\left(\int_{0}^{1}w^{2}u^{\frac{2}{{\mathrm{ID}}_{F}^{*}}}-2w^{2}u^{\frac{1}{{\mathrm{ID}}_{F}^{*}}+\frac{1}{{\mathrm{ID}}_{G}^{*}}}+w^{2}u^{\frac{2}{{\mathrm{ID}}_{G}^{*}}}\;du\right)}^{\frac{1}{2}}\\ & = & \lim_{w\to 0^{+}}w\cdot {\left(\frac{1}{\frac{2}{{\mathrm{ID}}_{F}^{*}}+1}-\frac{2}{\frac{1}{{\mathrm{ID}}_{F}^{*}}+\frac{1}{{\mathrm{ID}}_{G}^{*}}+1}+\frac{1}{\frac{2}{{\mathrm{ID}}_{G}^{*}}+1}\right)}^{\frac{1}{2}}\;=\;0\;. \end{array}$$

### 6.5. Normalization

Even though the limit of the second tail Wasserstein distance is zero and therefore uninteresting, we observe that by normalizing it by the tail length *w*, we arrive at a more useful result:

$$\begin{array}{ccc} \lim_{w\to 0^{+}}\frac{1}{w}{\mathrm{WD}}_{2}\left(F;G,w\right) & = & {\left(\frac{1}{\frac{2}{{\mathrm{ID}}_{F}^{*}}+1}-\frac{2}{\frac{1}{{\mathrm{ID}}_{F}^{*}}+\frac{1}{{\mathrm{ID}}_{G}^{*}}+1}+\frac{1}{\frac{2}{{\mathrm{ID}}_{G}^{*}}+1}\right)}^{\frac{1}{2}}\;. \end{array}$$

In general, reweighting by a power of *w* may be required to expose a relationship between the tail limit of an entropy measure or divergence and an expression in terms of the local intrinsic dimensions of the functions involved. Since local intrinsic dimension is a unitless quantity, in order to establish a non-trivial formulation solely in terms of LID values, any tail measure whose values are not unitless will generally require some form of normalization.

### 6.6. Summary of Results

Table 1 provides a summary of results. All the results stated in Table 1 can be derived either using the techniques outlined earlier in this section, or through direct substitution of another result in the table. The derivations are outlined in Table 2 (tail entropy variants), Table 3 (tail divergence variants), Table 4 (tail distance variants) and Table 5 (tail Wasserstein distances). Most of these derivations are straightforward; however, for two of the tail measures, some clarifications are required.

Generally speaking, for the normalized tail Wasserstein distances with *p* non-integer or *p* odd (Table 5), Lemma 2 cannot be applied, due to the absolute value operation in the integrand. It may happen that the functions $F^{-1}\left(u\right)$ and $G^{-1}\left(u\right)$ may have crossing points for many (possibly even infinitely many) values of *u* between 0 and 1. At these values of *u*, $\left|F^{-1}\left(u\right)-G^{-1}\left(u\right)\right|=0$, and neither $\left|z-G^{-1}\left(u\right)\right|$ nor $\left|F^{-1}\left(u\right)-y\right|$ would be monotone in the vicinity of $z=F^{-1}\left(u\right)$ or $y=G^{-1}\left(u\right)$, as the case may be.

For the tail JS divergence (Table 3), the derivation relies on the fact that the LID of the sum (or average) of two non-negative smooth growth functions is the smaller of the two individual LID values. This is an implication of the fact that $\lim_{t\to 0^{+}}\frac{V(t)}{W(t)}=0$ whenever the smooth growth functions V(t) and W(t) have $0<{\mathrm{ID}}_{W}^{*}<{\mathrm{ID}}_{V}^{*}$ (see [84] for more details). Accordingly, if ${\mathrm{ID}}_{F}^{*}\neq {\mathrm{ID}}_{G}^{*}$, then the function (*F* or *G*) with smaller LID value must have the same LID value as the average function $M\left(t\right)=\frac{F(t)+G(t)}{2}$, and the other function (*G* or *F*) must have LID value equal to the maximum of the two. From these observations, the derivation can be seen to hold.

The result for the limit of the tail KL divergence has an interesting interpretation in light of the so-called Itakura–Saito (IS) divergence (or distance) [85]:

$$d_{\mathrm{IS}}\left(x|y\right)\;=\;\sum_{i=1}^{n}\left(\frac{x_{i}}{y_{i}}-\ln\frac{x_{i}}{y_{i}}-1\right)\;.$$

As the tail boundary *w* tends to 0, the tail KL divergence between smooth functions *F* and *G* tends to the (univariate) IS divergence between their associated LID values ${\mathrm{ID}}_{G}^{*}$ and ${\mathrm{ID}}_{F}^{*}$:

$$\lim_{w\to 0^{+}}\mathrm{KL}\left(F;G,w\right)\;=\;\frac{{\mathrm{ID}}_{G}^{*}}{{\mathrm{ID}}_{F}^{*}}-\ln\frac{{\mathrm{ID}}_{G}^{*}}{{\mathrm{ID}}_{F}^{*}}-1\;=\;d_{\mathrm{IS}}\left({\mathrm{ID}}_{G}^{*}|{\mathrm{ID}}_{F}^{*}\right)\;.$$

When *F* and *G* are interpreted as the CDFs of distance distributions, the shape parameters of the extreme-value-theoretic generalized pareto distributions (GPDs) that asymptotically characterize their lower tails are known to equal $-\frac{1}{{\mathrm{ID}}_{F}^{*}}$ and $-\frac{1}{{\mathrm{ID}}_{G}^{*}}$, respectively [40]. Since the ratio of these parameters is equal to (the reciprocal of) the ratio of LID values, the tail KL divergence between *F* and *G* can also be interpreted as tending to the IS divergence between GPD parameters.

The IS divergence is popular as an objective for matrix factorization of audio spectra [86], for assessing the loss of using entry $y_{i,j}$ to approximate a true entry $x_{i,j}$; more precisely, to approximate a matrix V by factorization WH, the loss is $\sum_{i}\sum_{j}d_{\mathrm{IS}}\left({\left[V\right]}_{ij}|{\left[\mathrm{WH}\right]}_{ij}\right)$. The IS divergence is a convenient choice for this scenario due to its scale-free property ($d_{\mathrm{IS}}\left(x|y\right)=d_{\mathrm{IS}}\left(\alpha x|\alpha y\right)$ for any α≠0), thus giving the same relative weight to both small and large values of $x_{i}$ and $y_{i}$, since they only appear as the ratio $\frac{x_{i}}{y_{i}}$. This is important for scenarios such as audio spectra, where the magnitudes of $x_{i}$ and $y_{i}$ can vary greatly.

The Itakura–Saito divergence falls into the family of so-called Bregman divergences (or distances) [87], which have a geometric interpretation as the difference between the value of a convex generator function at x on the one hand, and the value at x of a hyperplane function that is tangent to the generator curve at y. Bregman divergences are a highly expressive family of distances with a wide range of applications [88]. For the IS divergence, the convex generator function is the negative logarithm $-\sum_{i=1}^{n}\ln x_{i}$. Interestingly, the KL divergence is also a Bregman divergence, with its convex generator being the negative entropy function $\sum_{i=1}^{n}x_{i}\ln x_{i}$ [89].

## 7. Extension to Multivariate Distributions

Thus far, our results have focused on a univariate scenario, wherein entropy and divergence variants were shown to be asymptotically equivalent to formulations involving the local intrinsic dimensionalities of smooth distributions of a single random variable. As discussed in Section 3, these results can be applied to distance-based analysis, through characterizations involving the LIDs of local (univariate) distance distributions induced by the overall (global) multivariate distribution. These characterizations are indirect, in that they do not explicitly involve (nor do they require) any knowledge of the underlying global distribution and its parameters. However, characterizations in terms of induced distance distributions may not be entirely satisfying when the nature of the global multivariate distribution is either known or assumed. In this section, we will assume that our domain S is the *n*-dimensional space $R^{n}$ equipped with the Euclidean distance, d(x,y)=∥x−y∥. Within S, we will also assume that we are given a data distribution D with probability density function $p:R^{n}\to R_{+}\cup 0$.

### 7.1. Multivariate Tail Distributions with Local Spherical Symmetry

Within the Euclidean domain, the challenge is to analyze distributions in terms of the probability measure captured within volumes associated with a distributional tail. However, unlike in univariate distributions, there is no universally accepted notion of ‘distributional tail’ for multivariate distributions. For our purposes, given a distance r>0, we define the tail of D of length *r* to be the region enclosed by the ball of radius *r* centered at the origin; that is, $B\left(r\right)≜\{x\in R^{n}:∥x∥\leq r\}$. The boundary of the tail is the (n−1)-dimensional surface area of B(r), which we denote by $B^{\prime }\left(r\right)≜\{x\in R^{n}:∥x∥=r\}$.

To enable tractable analysis, we will assume that the PDF can be expressed in terms of a locally spherically symmetrical function. One example of where local spherical symmetry can be expected to hold is for a locally isotropic context. This is a common assumption for physical systems, including metals, glasses, fluids and polymers, for which the distribution locally surrounding a particle in the system does not have a directional preference.

Formally, we say that a density function *f* is locally spherically symmetrical within radius *w* if for all ∥x∥≤w, we have $f\left(x\right)=f_{*}\left(r\right)$ for some univariate function $f_{*}$ where r=∥x∥. For *f* to be locally spherically symmetrical, it suffices that f(x) be equal to f(y) whenever 0≤∥x∥=∥y∥≤r. The assumption also implies the existence of a function $f_{*}$ for which $f\left(x\right)=f_{*}\left(r\right)$, and therefore that *f* must be constant over all points of the sphere $B^{\prime }\left(r\right)$.

The probability measure captured by B(r), which we denote by F(r), is obtained through the integration of *f* over this ball:

$$F\left(r\right)\;≜\;\int_{B(r)}f\;dB\left(r\right)\;.$$

It is not difficult to see that the univariate function *F* is simply the CDF of the distribution of distances to the origin induced by the global distribution D. If *F* is differentiable over the tail interval (0,r], then the integral of $F^{\prime }$ over this interval exists, and equals *F*:

(2) $$\begin{array}{ccc} \int_{B(r)}f\;dB\left(r\right) & =\;F(r)=\; & \int_{0}^{r}F^{\prime }\left(t\right)\;dt\;. \end{array}$$

The derivative $F^{\prime }\left(r\right)$ can therefore be interpreted as the PDF of the radial distance distribution as measured from the origin.

For spherically symmetric distributions in Euclidean spaces, the multivariate density and radial density is related through a factor that depends on the surface area of spherical volumes. The formulae for the volume of an *n*-sphere and its (n−1)-dimensional surface area are given by

$$\begin{array}{ccc} V_{n}\left(r\right) & ≜ & \frac{\pi^{n/2}}{\Gamma ((n/2)+1)}r^{n}\;\;\;\mathrm{and}\\ S_{n-1}\left(r\right) & ≜ & \frac{2\pi^{n/2}}{\Gamma (n/2)}r^{n-1}\;, \end{array}$$

respectively. Γ is the common gamma function Γ(n)=(n−1)! if *n* is a positive integer and $\Gamma \left(n+\frac{1}{2}\right)=\left(n-\frac{1}{2}\right)\left(n-\frac{3}{2}\right)\ldots \frac{1}{2}\sqrt{\pi }$ if *n* is a non negative integer. Furthermore, the volume and surface area have a simple relationship that allows for easy conversion between the two:

(3) $$r\cdot S_{n-1}\left(r\right)\;=\;n\cdot V_{n}\left(r\right)\;\;.$$

**Lemma** **4** ([90])**.** *Let X be an n-dimensional random vector that is spherically symmetric with a radial distribution R. Then X has a density f(x) if and only if R has a density s and*

$$s\left(r\right)=f\left(x\right)\cdot S_{n-1}\left(r\right)\;.$$

If *F* is a smooth growth function that is locally spherically symmetric over [0,r], Equation (2) and Lemma 4 together give us the following relationship between the radial density $F^{\prime }$ and the multivariate density *f*:

$$f\left(x\right)\;=\;\frac{F^{\prime }\left(∥x∥\right)}{S_{n-1}\left(∥x∥\right)}$$

whenever ∥x∥≤r. Conditioning the distribution to the ball B(r), the tail distribution PDF becomes

$$f_{r}\left(x\right)\;≜\;\frac{f(x)}{\int_{B(w)}f\;dB\left(w\right)}\;=\;\frac{F^{\prime }\left(∥x∥\right)}{S_{n-1}\left(∥x∥\right)\cdot F\left(r\right)}\;=\;\frac{F_{r}^{\prime }\left(∥x∥\right)}{S_{n-1}\left(∥x∥\right)}\;.$$

### 7.2. Multivariate Tail Entropy Variants

The aforementioned relationships between multivariate and radial densities can be immediately used to compute the various tail entropies for the locally spherically symmetric multivariate case. Useful background on evaluating radial integrals can be found in Baker [91]. For example, the multivariate Tail Entropy is

$$\begin{array}{ccc} H(f,w) & ≜ & -\int_{B(w)}f_{w}\ln f_{w}\;dB\left(w\right)\\ & = & -\int_{0}^{w}\left(\frac{F_{w}^{\prime }\left(t\right)}{S_{n-1}\left(t\right)}\ln\frac{F_{w}^{\prime }\left(t\right)}{S_{n-1}\left(t\right)}\right)\cdot S_{n-1}\left(t\right)\;dt\\ & = & -\int_{0}^{w}F_{w}^{\prime }\left(t\right)\ln\frac{F_{w}^{\prime }\left(t\right)}{S_{n-1}\left(t\right)}\;dt\;. \end{array}$$

Although the multivariate formulation of Tail Entropy H(f,w) resembles that of the univariate formulation H(F,w), the two are not identical. Nevertheless, the multivariate formulation can still be simplified using the technical lemmas introduced in Section 5. In much the same way as for the univariate Tail Entropy Power, we can use Theorem 1 together with Lemmas 1 and 3 to determine the limit of H(f,w) as *w* tends to 0. Replacing $F_{w}^{\prime }\left(t\right)$ by $\frac{1}{t}{\mathrm{ID}}_{F}\left(t\right)F_{w}\left(t\right)$, then ${\mathrm{ID}}_{F}\left(t\right)$ by ${\mathrm{ID}}_{F}^{*}$, and finally $F_{w}\left(t\right)$ by ${\left(\frac{t}{w}\right)}^{{\mathrm{ID}}_{F}^{*}}$, we obtain

$$\begin{array}{ccc} \lim_{w\to 0}H\left(f,w\right) & = & \lim_{w\to 0}-\int_{0}^{w}F_{w}^{\prime }\left(t\right)\ln\frac{F_{w}^{\prime }\left(t\right)}{t^{n-1}S_{n-1}\left(1\right)}\;dt\\ & = & \lim_{w\to 0}-\int_{0}^{w}\frac{{\mathrm{ID}}_{F}^{*}}{t}{\left(\frac{t}{w}\right)}^{{\mathrm{ID}}_{F}^{*}}\ln\left[\frac{{\mathrm{ID}}_{F}^{*}}{t^{n}S_{n-1}\left(1\right)}{\left(\frac{t}{w}\right)}^{{\mathrm{ID}}_{F}^{*}}\right]\;dt\\ & = & \lim_{w\to 0}-\int_{0}^{w}\frac{{\mathrm{ID}}_{F}^{*}}{w^{{\mathrm{ID}}_{F}^{*}}}t^{{\mathrm{ID}}_{F}^{*}-1}\ln\left[\frac{{\mathrm{ID}}_{F}^{*}}{w^{{\mathrm{ID}}_{F}^{*}}S_{n-1}\left(1\right)}t^{{\mathrm{ID}}_{F}^{*}-n}\right]\;dt\\ & = & \lim_{w\to 0}-\int_{0}^{w}\frac{{\mathrm{ID}}_{F}^{*}}{w^{{\mathrm{ID}}_{F}^{*}}}t^{{\mathrm{ID}}_{F}^{*}-1}\left[\ln\frac{{\mathrm{ID}}_{F}^{*}}{w^{{\mathrm{ID}}_{F}^{*}}S_{n-1}\left(1\right)}+\left({\mathrm{ID}}_{F}^{*}-n\right)\ln t\right]\;dt\;. \end{array}$$

Solving the integral, and then using Equation (3) to convert the surface area factor $S_{n-1}$ to an expression involving the volume $V_{n}$, we eventually arrive at

$$\begin{array}{ccc} \lim_{w\to 0}H\left(f,w\right) & = & \lim_{w\to 0}\left(1-\frac{n}{{\mathrm{ID}}_{F}^{*}}-\ln\frac{{\mathrm{ID}}_{F}^{*}}{w^{n}\;S_{n-1}\left(1\right)}\right)\\ & = & \lim_{w\to 0}\left(1-\frac{n}{{\mathrm{ID}}_{F}^{*}}-\ln\frac{{\mathrm{ID}}_{F}^{*}}{w\;S_{n-1}\left(w\right)}\right)\\ & = & \lim_{w\to 0}\left(1-\frac{n}{{\mathrm{ID}}_{F}^{*}}-\ln\frac{{\mathrm{ID}}_{F}^{*}}{n}+\ln V_{n}\left(w\right)\right)\;, \end{array}$$

which diverges even when the Tail Entropy is reweighted by $V_{n}\left(w\right)$ (or indeed, by any other polynomial in *w*). However, the Tail Entropy Power, when normalized by $V_{n}\left(w\right)$, does converge to a strictly positive value:

$$\begin{array}{ccc} \lim_{w\to 0}\frac{1}{V_{n}\left(w\right)}\mathrm{HP}\left(f,w\right) & ≜ & \lim_{w\to 0}\frac{1}{V_{n}\left(w\right)}\exp\left(H(f,w)\right)\\ & = & \lim_{w\to 0}\frac{1}{V_{n}\left(w\right)}\exp\left(1-\frac{n}{{\mathrm{ID}}_{F}^{*}}-\ln\frac{{\mathrm{ID}}_{F}^{*}}{n}+\ln V_{n}\left(w\right)\right)\\ & = & \frac{1}{\varphi }\exp\left(1-\frac{1}{\varphi }\right),\;\;\;\mathrm{where}\;\;\varphi =\frac{{\mathrm{ID}}_{F}^{*}}{n}\;. \end{array}$$

As one might expect in the *n*-dimensional Euclidean setting, the (normalized asymptotic) multivariate Tail Entropy Power is maximized whenever ${\mathrm{ID}}_{F}^{*}$, the local intrinsic dimensionality of the associated radial CDF *F*, is equal to *n*.

### 7.3. Multivariate Cumulative Tail Entropy

In the multivariate setting, cumulative entropy is defined in terms of the distributional tail, according to the notion laid out in Section 7.1. In place of the usual probability density f(x), the entropy function is applied to the probability measure associated with the ball centered at the origin with radius ∥x∥; that is, with

$$\mathrm{Pr}\left[X\leq ∥x∥\right]\;≜\;\int_{B(∥x∥)}f\;dB\left(∥x∥\right)\;=\;F\left(∥x∥\right)\;.$$

Note that since *F* takes the same value at x and y whenever ∥x∥=∥y∥, the quantity F(∥x∥) is locally spherically symmetric even when the underlying density function *f* is not.

We can adapt the multivariate formulation of cumulative residual entropy that was originally proposed by Rao [56]. The multivariate Cumulative Tail Entropy, conditioned to a distributional tail of radius *w*, is expressed as a multivariate integral involving $F_{w}\left(∥x∥\right)$, or as a radial integral involving $F_{w}$, as follows:

$$\begin{array}{ccc} \mathrm{cH}(f,w) & ≜ & -\int_{x\in B(w)}F_{w}\left(∥x∥\right)\;\ln F_{w}\left(∥x∥\right)\;dB\left(w\right)\\ & = & -\int_{0}^{w}\left(F_{w}\left(t\right)\;\ln F_{w}\left(t\right)\right)\cdot S_{n-1}\left(t\right)\;dt\;. \end{array}$$

As in the treatment of the univariate tail entropies, we can use Lemma 1 to determine the limit of cH(f,w) as *w* tends to 0. Replacing $F_{w}\left(t\right)$ by ${\left(\frac{t}{w}\right)}^{{\mathrm{ID}}_{F}^{*}}$,

$$\begin{array}{ccc} \lim_{w\to 0}\mathrm{cH}\left(f,w\right) & = & \lim_{w\to 0}-\int_{0}^{w}t^{n-1}S_{n-1}\left(1\right)\;F_{w}\left(t\right)\;\ln F_{w}\left(t\right)\;dt\\ & = & \lim_{w\to 0}-\int_{0}^{w}t^{n-1}S_{n-1}\left(1\right)\;{\left(\frac{t}{w}\right)}^{{\mathrm{ID}}_{F}^{*}}\;\ln{\left(\frac{t}{w}\right)}^{{\mathrm{ID}}_{F}^{*}}\;dt\\ & = & \lim_{w\to 0}-\int_{0}^{w}\frac{S_{n-1}\left(1\right)\;{\mathrm{ID}}_{F}^{*}}{w^{{\mathrm{ID}}_{F}^{*}}}\cdot t^{{\mathrm{ID}}_{F}^{*}+n-1}\;\left(\ln t-\ln w\right)\;dt\;. \end{array}$$

Solving the integral, and then converting the surface area factor $S_{n-1}$ to a volume factor $V_{n}$ using Equation (3), we obtain

$$\begin{array}{ccc} \lim_{w\to 0}\mathrm{cH}\left(f,w\right) & = & \lim_{w\to 0}w\;S_{n-1}\left(w\right)\cdot \frac{{\mathrm{ID}}_{F}^{*}}{{\left({\mathrm{ID}}_{F}^{*}+n\right)}^{2}}\\ & = & \lim_{w\to 0}V_{n}\left(w\right)\cdot \frac{\varphi }{{\left(\varphi +1\right)}^{2}}\;,\;\;\;\mathrm{where}\;\;\varphi =\frac{{\mathrm{ID}}_{F}^{*}}{n}\;. \end{array}$$

Although the multivariate Cumulative Tail Entropy vanishes as the tail boundary *w* tends to zero, when normalized by the tail volume $V_{n}\left(w\right)$ it converges to a strictly positive value:

$$\begin{array}{ccc} \lim_{w\to 0}\frac{1}{V_{n}\left(w\right)}\mathrm{cH}\left(f,w\right) & = & \frac{\varphi }{{\left(\varphi +1\right)}^{2}}\;. \end{array}$$

Again, as with the Normalized Tail Entropy Power, the (asymptotic) multivariate Tail Cumulative Entropy is maximized whenever φ=1. That is, when ${\mathrm{ID}}_{F}^{*}=n$.

### 7.4. Multivariate Tail Divergences

Several of the tail divergence measures, when considered in the multivariate setting under the assumptions of locally spherical symmetry, turn out to be identical to those of the radial (univariate) setting. As an example, consider the multivariate Tail KL Divergence, defined as

$$\begin{array}{ccc} \mathrm{KL}(f;g,w) & ≜ & \int_{B(w)}f_{w}\ln\frac{f_{w}}{g_{w}}\;dB\left(w\right)\;. \end{array}$$

Applying Lemma 4 and integrating radially over the tail, we see that

$$\begin{array}{ccc} \mathrm{KL}(f;g,w) & = & \int_{0}^{w}\left(\frac{F_{w}^{\prime }\left(t\right)}{S_{n-1}\left(t\right)}\ln\frac{F_{w}^{\prime }\left(t\right)/S_{n-1}\left(t\right)}{G_{w}^{\prime }\left(t\right)/S_{n-1}\left(t\right)}\right)\cdot S_{n-1}\left(t\right)\;dt\\ & = & \int_{0}^{w}F_{w}^{\prime }\left(t\right)\ln\frac{F_{w}^{\prime }\left(t\right)}{G_{w}^{\prime }\left(t\right)}\;dt\\ & = & \mathrm{KL}(F;G,w)\;, \end{array}$$

the Tail KL Divergence of *F* and *G*, which (as stated in Table 1) has the limit $\frac{{\mathrm{ID}}_{G}^{*}}{{\mathrm{ID}}_{F}^{*}}-\ln\frac{{\mathrm{ID}}_{G}^{*}}{{\mathrm{ID}}_{F}^{*}}-1$ as the tail length *w* tends to zero.

Similarly, it can easily be seen that the multivariate versions of the JS Divergence, the Hellinger Distance, the $\chi^{2}$-Divergence and the α-Divergence all have radial integral formulations identical to their corresponding univariate versions.

### 7.5. Observations

The general strategy for deriving these results is essentially the same as for the multivariate Tail Entropy: first use Lemma 4 to convert the multidimensional integral to an integral in one dimension, then use the technical lemmas of Section 5 to simplify the univariate integral as before.

Our results for the locally spherically symmetric multivariate case are shown in Table 6; however, since their derivations greatly resemble those of the analogous univariate cases, we omit the details. Some remarks:

1. A result for the Wasserstein Distance is not included, since its formulation does not generalize straightforwardly to higher dimensions, unlike the other divergence measures.
2. The normalizations and weightings used depend only on the tail volume $V_{n}\left(w\right)$ and (for the Tsallis entropy variants) the parameter *q*. This generalizes our earlier univariate results where normalization was performed with regard to the tail length *w*.
3. All the multivariate tail variants considered Table 6 are elegant generalizations of their corresponding univariate formulations, and all explicitly depend on the ratios between the LIDs and the dimension of the space *n* ($\varphi =\frac{{\mathrm{ID}}_{F}^{*}}{n}$ and $\gamma =\frac{{\mathrm{ID}}_{G}^{*}}{n}$), or on the ratio of two LID values ($\rho =\frac{{\mathrm{ID}}_{G}^{*}}{{\mathrm{ID}}_{F}^{*}}=\frac{\gamma }{\varphi }$). Among these, the Normalized Entropy Power and the Normalized Cumulative Entropy are maximized when ${\mathrm{ID}}_{F}^{*}=n$, which can occur when the tail distribution is uniform. The Varentropy is minimized when ${\mathrm{ID}}_{F}^{*}=n$, which can occur when the variance of the log-likelihood for a uniform distribution is equal to zero.
4. As mentioned in Related Work, a number of previous studies in deep learning have found that the local intrinsic dimension in learned representations is lower than the dimension of the full space [32,33,34,35] (i.e., ${\mathrm{ID}}_{F}^{*}<n$) and that the learning process progressively reduces local intrinsic dimension. Consider a concrete example where n=100 and ${\mathrm{ID}}_{F}^{*}=12$ and the learning process is reducing ${\mathrm{ID}}_{F}^{*}$ at a point from 12 to 11. The consequent effect on entropy can be interpreted from two different perspectives, either as an increase in tail distance entropy or a decrease in tail location entropy:
  - Considering univariate normalized entropy power or normalized cumulative entropy (Table 1), reduction of ${\mathrm{ID}}_{F}^{*}$ corresponds to an increase in entropy. Here, the entropy is measuring the uncertainty of the univariate random variable modeling distances to nearest neighbors. Thus, reduction of ${\mathrm{ID}}_{F}^{*}$ corresponds to an increase in “distance entropy”.
  - Considering multivariate normalized entropy power or multivariate normalized cumulative entropy (Table 6), reduction of ${\mathrm{ID}}_{F}^{*}$ corresponds to an decrease in entropy. Here, the entropy is measuring the uncertainty of the multivariate random variable modeling locations of nearest neighbors, assuming local spherical symmetry. So reduction of ${\mathrm{ID}}_{F}^{*}$ corresponds to a decrease in “location entropy”.

We will see a visualization of these scenarios in Section 7.6.

5. All four of the multivariate tail divergences listed in Table 6, as well as the Hellinger Distance, have radial integral formulations that are identical to their univariate counterparts. All the divergences and distances (including the Weighted L2 Distance) are minimized when ${\mathrm{ID}}_{F}^{*}={\mathrm{ID}}_{G}^{*}$.
6. By setting n=1, we can recover the univariate results from Table 1. However, note that the range of integration used in Table 6 is a hypersphere of radius *w*, where for n=1 it is the interval [−w,w]. In contrast, the integral formulations listed in Table 1 were taken over the interval [0,w]. For some results, this means a minor (constant factor of 2) difference between Table 1 and the result from Table 6 when n=1.

### 7.6. Visualization of Behavior

**Figure 1:**
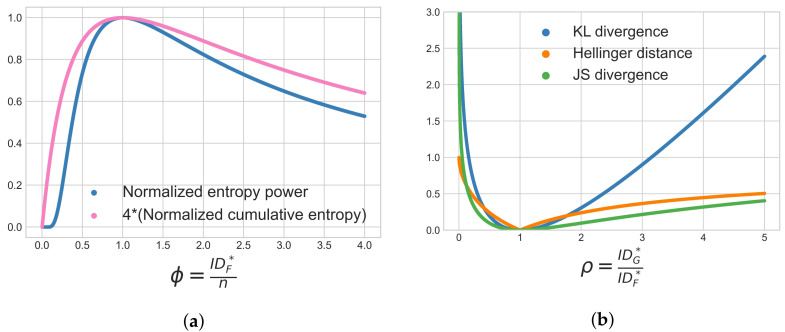
Visualization of selected measures from Table 6 (**a**) Entropy behavior as the ratio $\frac{{\mathrm{ID}}_{F}^{*}}{n}$ varies; (**b**) Divergence/distance behavior as the ratio $\frac{{\mathrm{ID}}_{G}^{*}}{{\mathrm{ID}}_{F}^{*}}$ varies.

Our results in Table 6 relate local intrinsic dimensionality to entropies and divergences. If analyzing an *n* dimensional global distribution such as the standard normal distribution or uniform distribution, then the dimension of every sub-manifold (i.e., the local intrinsic dimensionality ${\mathrm{ID}}_{F}^{*}$) will be *n*. However, our interest is in situations where the local intrinsic dimensionality differs from the representation dimension *n*. To provide further intuition on this aspect, two plots are shown in Figure 1.

Figure 1a compares the behavior of the normalized entropy power and the normalized cumulative entropy (multiplied by a constant factor of 4) in *n*-dimensional space, as the ratio $\phi =\frac{{\mathrm{ID}}_{F}^{*}}{n}$ is varied. We see that these measures have similar trends and they are maximized when ${\mathrm{ID}}_{F}^{*}=n$. We also see that when $1\ll {\mathrm{ID}}_{F}^{*}<n$, these entropic measures will decrease if ${\mathrm{ID}}_{F}^{*}$ is decreased (for a fixed *n*). On the other hand, if n=1 and $1\ll {\mathrm{ID}}_{F}^{*}$, then these entropic measures will increase if ${\mathrm{ID}}_{F}^{*}$ is decreased, where n=1 corresponds to the scenario where we are modeling the uncertainty of a distance distribution. This illustrates remark number 4 from Section 7.5 above.

Figure 1b compares the behavior of different tail divergences as the ratio $\rho =\frac{{\mathrm{ID}}_{G}^{*}}{{\mathrm{ID}}_{F}^{*}}$ varies. The divergences shown are the KL divergence, the Jensen–Shannon divergence and the Hellinger distance. These measures have similar trends as ρ varies and are minimized and equal to zero when ${\mathrm{ID}}_{F}^{*}={\mathrm{ID}}_{G}^{*}$. Also, the Hellinger distance is bounded above by 1.

## 8. Conclusions

In this theoretical investigation, we have established asymptotic relationships between tail entropy variants, tail divergences and the theory of local intrinsic dimensionality. Our results are derived under the assumption that the distribution(s) under consideration are being analyzed in a highly local context, within the distribution tail(s), an asymptotically small neighborhood whose radius approaches zero. These results show that tail entropies and tail divergences depend in a fundamental way on local intrinsic dimensionality and help form a theoretical foundation for cross-fertilization between intrinsic dimensionality research and entropy research. As future work, we plan to investigate the potential of these new characterizations in a range of application settings. For example, for use as a basis in machine learning to characterize and improve representations and representation learning, as well as use in understanding behavior of physical systems such as fluids and helping characterize their critical transitions in time and space.

Our results from both univariate and multivariate cases, show that the tail entropies and divergences considered in this paper depend only on (i) the embedding (representation) dimension in which the distribution is situated, and (ii) the local intrinsic dimension(s) of the distribution(s). Furthermore, in many cases there is dependence involving the ratio between the intrinsic dimension and the embedding dimension.

Consider the context of distance based analysis, when a distribution models distances from a central query location to its nearest neighbors, and the distances are induced by global data. In this situation, our characterization of entropy might be termed as ‘personalized’, in that entropy expresses the uncertainty (or complexity) from the perspective of the query, in regard to the distances to samples within an asymptotically small neighborhood. Phrased another way, these local entropies are ‘observer-dependent’, since they are tied to the choice of query (the observer). This can be contrasted with the more common notion of entropy, where one analyzes a global distribution, and there is no requirement of a query point or its local neighborhood.

As alluded to in the introduction, divergences between tail distributions could be used for comparison of real and synthetic distributions, as is commonly required for generative adversarial networks (GANs). Given a particular query location we may either: (i) compute the divergence between the univariate tail distance distributions of synthetic and real examples, as measured from a query point; or (ii) compute the divergence between the multivariate tail distance distributions of synthetic and real examples, again as measured from the query, under an assumption of local isotropy. Our results show that under the assumption of local spherical symmetry, the use of divergences (such as KL) between tail distance distributions is asymptotically equivalent to the standard multivariate formulations with the same divergences, when restricted to the neighborhoods around locations of interest. For future work it will be interesting to consider whether it is possible to further extend our multivariate results to elliptically symmetric distributions or skew-elliptical distributions, such as those studied by Contreras-Reyes [65].

Lastly, our results in Table 1 and Table 6 show theoretical relationships for entropies and divergences, but in practice one must estimate the measures using samples of data. A natural approach here is to first estimate local intrinsic dimensional values such as ${\mathrm{ID}}_{F}^{*}$ and ${\mathrm{ID}}_{G}^{*}$ using any desired estimator (such as the maximum likelihood estimator [39,40,41]), and then plug in the estimated LID value into the desired tail entropy or tail divergence formula. For example, an estimator of the (univariate) Normalized Cumulative Entropy could be obtained by computing $\frac{\hat{{\mathrm{ID}}_{F}^{*}}}{{\left(\hat{{\mathrm{ID}}_{F}^{*}}+1\right)}^{2}}$, where $\hat{{\mathrm{ID}}_{F}^{*}}$ is the estimated LID of the distance distribution *F*. 

## Figures and Tables

**Table 1:** Asymptotic equivalences between LID formulations and tail measures of entropy or divergence. In each case, the functions *F* and *G* are assumed to be smooth growth functions. In addition, for the Normalized Wasserstein Distance, *F* and *G* must be strictly monotonically increasing, thereby guaranteeing that the inverses of $F_{w}$ and $G_{w}$ exist near zero. In some cases, for the asymptotic limit to exist non-trivially (that is, to be both finite and non-zero), the tail entropy or tail divergence must be normalized by the multiplicative factor $\frac{1}{w}$, *w*. For the Tail Entropy and Tail Cross Entropy, no reweighting by powers of *w* can lead to a non-trivial asymptotic limit as *w* tends to zero.

Tail Measure	Formulation	Limit as $w\to 0^{+}$
Entropy	H(F,w) = $-\int_{0}^{w}F_{w}^{\prime }\left(t\right)\ln F_{w}^{\prime }\left(t\right)\;dt$	Diverges (no reweighting possible)
Varentropy	VarH(F,w) = $\int_{0}^{w}F_{w}^{\prime }\left(t\right)\ln^{2}F_{w}^{\prime }\left(t\right)\;dt\;-{\left(\int_{0}^{w}F_{w}^{\prime }\left(t\right)\ln F_{w}^{\prime }\left(t\right)\;dt\right)}^{2}$	${\left(1-\frac{1}{{\mathrm{ID}}_{F}^{*}}\right)}^{2}$
*q*-Entropy	$H_{q}\left(F,w\right)$ = $\frac{1}{q-1}\int_{0}^{w}F_{w}^{\prime }\left(t\right)-{\left(F_{w}^{\prime }\left(t\right)\right)}^{q}\;dt$	$\frac{1}{q-1}$ if q<1, diverges if q>1
Normalized Cumulative Entropy	$\frac{1}{w}\mathrm{cH}\left(F,w\right)$ = $-\frac{1}{w}\int_{0}^{w}F_{w}\left(t\right)\ln F_{w}\left(t\right)\;dt$	$\frac{{\mathrm{ID}}_{F}^{*}}{{\left({\mathrm{ID}}_{F}^{*}+1\right)}^{2}}$
Normalized Cumulative *q*-Entropy	$\frac{1}{w}{\mathrm{cH}}_{q}\left(F,w\right)$ = $\frac{1}{w(q-1)}\int_{0}^{w}F_{w}\left(t\right)-{\left(F_{w}\left(t\right)\right)}^{q}\;dt$	$\frac{{\mathrm{ID}}_{F}^{*}}{\left({\mathrm{ID}}_{F}^{*}+1\right)\left(q{\mathrm{ID}}_{F}^{*}+1\right)}$ if q≠1
Normalized Entropy Power	$\frac{1}{w}\mathrm{HP}\left(F,w\right)$ = $\frac{1}{w}\exp\left(H(F,w)\right)$	$\frac{1}{{\mathrm{ID}}_{F}^{*}}\exp\left(1-\frac{1}{{\mathrm{ID}}_{F}^{*}}\right)$
Normalized *q*-Entropy Power	$\frac{1}{w}{\mathrm{HP}}_{q}\left(F,w\right)$ = $\frac{1}{w}{\left[1+\left(1-q\right)\;H_{q}\left(F,w\right)\right]}^{\frac{1}{1-q}}$	${\left(\frac{{\left({\mathrm{ID}}_{F}^{*}\right)}^{q}}{q{\mathrm{ID}}_{F}^{*}-q+1}\right)}^{\frac{1}{1-q}}$
		if q≠1 and $q{\mathrm{ID}}_{F}^{*}-q+1>0$
Cross Entropy	XH(F;G,w) = $-\int_{0}^{w}F_{w}^{\prime }\left(t\right)\ln G_{w}^{\prime }\left(t\right)\;dt$	Diverges (no reweighting possible)
Normalized Cross Entropy Power	$\frac{1}{w}\mathrm{XHP}\left(F;G,w\right)$ = $\frac{1}{w}\exp\left({-\int }_{0}^{w}F_{w}^{\prime }\left(t\right)\ln G_{w}^{\prime }\left(t\right)\;dt\right)$	$\frac{1}{{\mathrm{ID}}_{G}^{*}}\exp\left(\frac{{\mathrm{ID}}_{G}^{*}-1}{{\mathrm{ID}}_{F}^{*}}\right)$
KL Divergence	KL(F;G,w) = $\int_{0}^{w}F_{w}^{\prime }\left(t\right)\ln\frac{F_{w}^{\prime }\left(t\right)}{G_{w}^{\prime }\left(t\right)}\;dt$	$\rho -\ln\rho -1;\;\;\;\rho =\frac{{\mathrm{ID}}_{G}^{*}}{{\mathrm{ID}}_{F}^{*}}$
JS Divergence	JS(F;G,w) = $\frac{1}{2}\left(\mathrm{KL}\left(F;\frac{F+G}{2},w\right)+\mathrm{KL}\left(G;\frac{F+G}{2},w\right)\right)$	$\frac{1}{2}\left(\tau -\ln\tau -1\right);\;\;\tau =\min\left\{\rho ,\frac{1}{\rho }\right\};\;\;\rho =\frac{{\mathrm{ID}}_{G}^{*}}{{\mathrm{ID}}_{F}^{*}}$
Weighted L2 Distance	wL2D(F;G,w) = $w\int_{0}^{w}{\left(F_{w}^{\prime }\left(t\right)-G_{w}^{\prime }\left(t\right)\right)}^{2}\;dt$	$\frac{{\left({\mathrm{ID}}_{F}^{*}-{\mathrm{ID}}_{G}^{*}\right)}^{2}}{2({\mathrm{ID}}_{F}^{*}+{\mathrm{ID}}_{G}^{*}-1)}\left[1+\frac{1}{\left(2{\mathrm{ID}}_{F}^{*}-1\right)\left(2{\mathrm{ID}}_{G}^{*}-1\right)}\right]$
		${\mathrm{ID}}_{F}^{*}>\frac{1}{2}$; ${\mathrm{ID}}_{G}^{*}>\frac{1}{2}$
Hellinger Distance	HD(F;G,w) = $\sqrt{\frac{1}{2}\int_{0}^{w}{\left(\sqrt{F_{w}^{\prime }\left(t\right)}-\sqrt{G_{w}^{\prime }\left(t\right)}\right)}^{2}\;dt}$	$\frac{\left|1-\sqrt{\rho }\right|}{\sqrt{1+\rho }};\;\;\;\rho =\frac{{\mathrm{ID}}_{G}^{*}}{{\mathrm{ID}}_{F}^{*}}$
$\chi^{2}$-Divergence	$\chi^{2}D\left(F;G,w\right)$ = $\int_{0}^{w}\frac{{\left(F_{w}^{\prime }\left(t\right)-G_{w}^{\prime }\left(t\right)\right)}^{2}}{G_{w}^{\prime }\left(t\right)}\;dt$	$\frac{{\left(1-\rho \right)}^{2}}{\rho (2-\rho )};\;\;\;\rho =\frac{{\mathrm{ID}}_{G}^{*}}{{\mathrm{ID}}_{F}^{*}};\;\rho <2$
α-Divergence	αD(F;G,w) = $\frac{1}{\alpha (1-\alpha )}\int_{0}^{w}\alpha F_{w}^{\prime }\left(t\right)+\left(1-\alpha \right)G_{w}^{\prime }\left(t\right)$	$\frac{1}{\alpha (1-\alpha )}\left(1-\frac{1}{\alpha \rho^{\alpha -1}+\left(1-\alpha \right)\rho^{\alpha }}\right)$
	$-{\left(F_{w}^{\prime }\left(t\right)\right)}^{\alpha }{\left(G_{w}^{\prime }\left(t\right)\right)}^{1-\alpha }\;dt$	$\rho =\frac{{\mathrm{ID}}_{G}^{*}}{{\mathrm{ID}}_{F}^{*}}\;;\;\alpha +\rho \left(1-\alpha \right)>0$
Normalized Wasserstein Distance	$\frac{1}{w}{\mathrm{WD}}_{p}\left(F;G,w\right)$ = $\frac{1}{w}{\left(\int_{0}^{1}{\left|F_{w}^{-1}\left(u\right)-G_{w}^{-1}\left(u\right)\right|}^{p}\;du\right)}^{\frac{1}{p}}$	p=2: $\sqrt{\frac{1}{\frac{2}{{\mathrm{ID}}_{F}^{*}}+1}-\frac{2}{\frac{1}{{\mathrm{ID}}_{F}^{*}}+\frac{1}{{\mathrm{ID}}_{G}^{*}}+1}+\frac{1}{\frac{2}{{\mathrm{ID}}_{G}^{*}}+1}}$
		*p* even: ${\left(\sum_{j=0}^{p}\frac{{\left(-1\right)}^{j}\left(\overset{p}{j}\right)}{\left(p-j\right)\cdot {\left({\mathrm{ID}}_{F}^{*}\right)}^{-1}+j\cdot {\left({\mathrm{ID}}_{G}^{*}\right)}^{-1}+1}\right)}^{\frac{1}{p}}$

**Table 2:** Derivations of asymptotic relationships between tail entropy variants and local intrinsic dimensionality. Each step shows the equivalences between the formulations when *w* is allowed to tend to zero. In the comments column, for each step of the derivation, the lemmas invoked are stated, as well as any additional assumptions made. If a normalization other weighting is needed to avoid divergence, or convergence to a constant (independent of *F*), the details are shown in a comment in the final step. In all cases, *F* is assumed to be a smooth growth function.

Tail Measure	Derivation Steps	Comments
Entropy	H(F,w) → $-\int_{0}^{w}F_{w}^{\prime }\left(t\right)\ln F_{w}^{\prime }\left(t\right)\;dt$	
	→ $-\int_{0}^{w}\frac{{\mathrm{ID}}_{F}\left(t\right)\;F_{w}\left(t\right)}{t}\ln\frac{{\mathrm{ID}}_{F}\left(t\right)\;F_{w}\left(t\right)}{t}\;dt$	using Theorem 1
	→ $-\int_{0}^{w}\frac{{\mathrm{ID}}_{F}^{*}\;F_{w}\left(t\right)}{t}\ln\frac{{\mathrm{ID}}_{F}^{*}\;F_{w}\left(t\right)}{t}\;dt$	using Lemma 3
	→ $-\int_{0}^{w}\frac{{\mathrm{ID}}_{F}^{*}}{t}{\left(\frac{t}{w}\right)}^{{\mathrm{ID}}_{F}^{*}}\ln\left[\frac{{\mathrm{ID}}_{F}^{*}}{t}{\left(\frac{t}{w}\right)}^{{\mathrm{ID}}_{F}^{*}}\right]\;dt$	using Lemma 1
	→ $1-\frac{1}{{\mathrm{ID}}_{F}^{*}}-\ln\frac{{\mathrm{ID}}_{F}^{*}}{w}$	no reweighting
Varentropy	VarH(F,w) → $\int_{0}^{w}F_{w}^{\prime }\left(t\right)\ln^{2}F_{w}^{\prime }\left(t\right)\;dt\;-{\left(\int_{0}^{w}F_{w}^{\prime }\left(t\right)\ln F_{w}^{\prime }\left(t\right)\;dt\right)}^{2}$	
	→ $\int_{0}^{w}\frac{{\mathrm{ID}}_{F}\left(t\right)\;F_{w}\left(t\right)}{t}\ln^{2}\frac{{\mathrm{ID}}_{F}\left(t\right)\;F_{w}\left(t\right)}{t}\;dt\;-{\left(\int_{0}^{w}\frac{{\mathrm{ID}}_{F}\left(t\right)\;F_{w}\left(t\right)}{t}\ln\frac{{\mathrm{ID}}_{F}\left(t\right)\;F_{w}\left(t\right)}{t}\;dt\right)}^{2}$	using Theorem 1
	→ $\int_{0}^{w}\frac{{\mathrm{ID}}_{F}^{*}\;F_{w}\left(t\right)}{t}\ln^{2}\frac{{\mathrm{ID}}_{F}^{*}\;F_{w}\left(t\right)}{t}\;dt\;-{\left(\int_{0}^{w}\frac{{\mathrm{ID}}_{F}^{*}\;F_{w}\left(t\right)}{t}\ln\frac{{\mathrm{ID}}_{F}^{*}\;F_{w}\left(t\right)}{t}\;dt\right)}^{2}$	using Lemma 3
	→ $\int_{0}^{w}\frac{{\mathrm{ID}}_{F}^{*}}{t}{\left(\frac{t}{w}\right)}^{{\mathrm{ID}}_{F}^{*}}\ln^{2}\left[\frac{{\mathrm{ID}}_{F}^{*}}{t}{\left(\frac{t}{w}\right)}^{{\mathrm{ID}}_{F}^{*}}\right]\;dt\;-{\left(\int_{0}^{w}\frac{{\mathrm{ID}}_{F}^{*}}{t}{\left(\frac{t}{w}\right)}^{{\mathrm{ID}}_{F}^{*}}\ln\left[\frac{{\mathrm{ID}}_{F}^{*}}{t}{\left(\frac{t}{w}\right)}^{{\mathrm{ID}}_{F}^{*}}\right]\;dt\right)}^{2}$	using Lemma 1
	→ ${\left(1-\frac{1}{{\mathrm{ID}}_{F}^{*}}\right)}^{2}$	
*q*-Entropy	$H_{q}\left(F,w\right)$ → $\frac{1}{q-1}\int_{0}^{w}F_{w}^{\prime }\left(t\right)-{\left(F_{w}^{\prime }\left(t\right)\right)}^{q}\;dt$	q>1
	→ $\frac{1}{q-1}\int_{0}^{w}\frac{{\mathrm{ID}}_{F}\left(t\right)\;F_{w}\left(t\right)}{t}-{\left(\frac{{\mathrm{ID}}_{F}\left(t\right)\;F_{w}\left(t\right)}{t}\right)}^{q}\;dt$	using Theorem 1
	→ $\frac{1}{q-1}\int_{0}^{w}\frac{{\mathrm{ID}}_{F}^{*}\;F_{w}\left(t\right)}{t}-{\left(\frac{{\mathrm{ID}}_{F}^{*}\;F_{w}\left(t\right)}{t}\right)}^{q}\;dt$	using Lemma 3
	→ $\frac{1}{q-1}\int_{0}^{w}\frac{{\mathrm{ID}}_{F}^{*}}{t}{\left(\frac{t}{w}\right)}^{{\mathrm{ID}}_{F}^{*}}-{\left(\frac{{\mathrm{ID}}_{F}^{*}}{t}{\left(\frac{t}{w}\right)}^{{\mathrm{ID}}_{F}^{*}}\right)}^{q}\;dt$	using Lemma 1
	→ $\frac{1}{q-1}\left(1-\frac{1}{w^{q-1}}\cdot \frac{{\left({\mathrm{ID}}_{F}^{*}\right)}^{q}}{q{\mathrm{ID}}_{F}^{*}-q+1}\right)$	
Cumulative Entropy	cH(F,w) → $-\int_{0}^{w}F_{w}\left(t\right)\ln F_{w}\left(t\right)\;dt$	
	→ $-\int_{0}^{w}{\left(\frac{t}{w}\right)}^{{\mathrm{ID}}_{F}^{*}}\ln{\left(\frac{t}{w}\right)}^{{\mathrm{ID}}_{F}^{*}}\;dt$	using Lemma 1
	→ $w\frac{{\mathrm{ID}}_{F}^{*}}{{\left({\mathrm{ID}}_{F}^{*}+1\right)}^{2}}$	weight by $\frac{1}{w}$
Cumulative *q*-Entropy	${\mathrm{cH}}_{q}\left(F,w\right)$ → $\frac{1}{q-1}\int_{0}^{w}F_{w}\left(t\right)-{\left(F_{w}\left(t\right)\right)}^{q}\;dt$	q≠1
	→ $\frac{1}{q-1}\int_{0}^{w}{\left(\frac{t}{w}\right)}^{{\mathrm{ID}}_{F}^{*}}-{\left(\frac{t}{w}\right)}^{q{\mathrm{ID}}_{F}^{*}}\;dt$	using Lemma 1
	→ $w\frac{{\mathrm{ID}}_{F}^{*}}{\left({\mathrm{ID}}_{F}^{*}+1\right)\left(q{\mathrm{ID}}_{F}^{*}+1\right)}$	weight by $\frac{1}{w}$
Entropy Power	HP(F,w) → $\exp\left(H(F,w)\right)$	
	→ $\exp\left(1-\frac{1}{{\mathrm{ID}}_{F}^{*}}-\ln\frac{{\mathrm{ID}}_{F}^{*}}{w}\right)$	by substitution
	→ $w\frac{1}{{\mathrm{ID}}_{F}^{*}}\exp\left(1-\frac{1}{{\mathrm{ID}}_{F}^{*}}\right)$	weight by $\frac{1}{w}$
*q*-Entropy Power	${\mathrm{HP}}_{q}\left(F,w\right)$ → ${\left[1+\left(1-q\right)\;H_{q}\left(F,w\right)\right]}^{\frac{1}{1-q}}$	q≠1
	→ ${\left(1+\left(1-q\right)\cdot \frac{1}{q-1}\left[1-\frac{1}{w^{q-1}}\cdot \frac{{\left({\mathrm{ID}}_{F}^{*}\right)}^{q}}{q{\mathrm{ID}}_{F}^{*}-q+1}\right]\right)}^{\frac{1}{1-q}}$	by substitution
	→ $w{\left(\frac{{\left({\mathrm{ID}}_{F}^{*}\right)}^{q}}{q{\mathrm{ID}}_{F}^{*}-q+1}\right)}^{\frac{1}{1-q}}$	weight by $\frac{1}{w}$

**Table 3:** Derivations of asymptotic relationships between tail divergences and local intrinsic dimensionality. Each step shows the equivalences between the formulations when *w* is allowed to tend to zero. In the comments column, for each step of the derivation, the lemmas invoked are stated, as well as any additional assumptions made. If a normalization or weighting is needed, the details are shown in a comment in the final step. In all cases, *F* and *G* are assumed to be smooth growth functions.

Tail Measure	Derivation Steps	Comments
Cross Entropy	XH(F;G,w) → $-\int_{0}^{w}F_{w}^{\prime }\left(t\right)\ln G_{w}^{\prime }\left(t\right)\;dt$	
	→ $-\int_{0}^{w}\frac{{\mathrm{ID}}_{F}\left(t\right)\;F_{w}\left(t\right)}{t}\ln\frac{{\mathrm{ID}}_{G}\left(t\right)\;G_{w}\left(t\right)}{t}\;dt$	using Theorem 1
	→ $-\int_{0}^{w}\frac{{\mathrm{ID}}_{F}^{*}\;F_{w}\left(t\right)}{t}\ln\frac{{\mathrm{ID}}_{G}^{*}\;G_{w}\left(t\right)}{t}\;dt$	using Lemma 3
	→ $-\int_{0}^{w}\frac{{\mathrm{ID}}_{F}^{*}}{t}{\left(\frac{t}{w}\right)}^{{\mathrm{ID}}_{F}^{*}}\ln\left[\frac{{\mathrm{ID}}_{G}^{*}}{t}{\left(\frac{t}{w}\right)}^{{\mathrm{ID}}_{G}^{*}}\right]\;dt$	using Lemma 1
	→ $\frac{{\mathrm{ID}}_{G}^{*}-1}{{\mathrm{ID}}_{F}^{*}}-\ln\frac{{\mathrm{ID}}_{G}^{*}}{w}$	no reweighting
Cross Entropy Power	XHP(F;G,w) → $\exp\left(\mathrm{XH}(F;G,w)\right)$	
	→ $\exp\left(\frac{{\mathrm{ID}}_{G}^{*}-1}{{\mathrm{ID}}_{F}^{*}}-\ln\frac{{\mathrm{ID}}_{G}^{*}}{w}\right)$	by substitution
	→ $w\frac{1}{{\mathrm{ID}}_{G}^{*}}\exp\left(\frac{{\mathrm{ID}}_{G}^{*}-1}{{\mathrm{ID}}_{F}^{*}}\right)$	weight by $\frac{1}{w}$
KL Divergence	KL(F;G,w) → $\int_{0}^{w}F_{w}^{\prime }\left(t\right)\ln\frac{F_{w}^{\prime }\left(t\right)}{G_{w}^{\prime }\left(t\right)}\;dt$	
	→ $\int_{0}^{w}\frac{{\mathrm{ID}}_{F}\left(t\right)\;F_{w}\left(t\right)}{t}\ln\frac{{\mathrm{ID}}_{F}\left(t\right)\;F_{w}\left(t\right)}{{\mathrm{ID}}_{G}\left(t\right)\;G_{w}\left(t\right)}\;dt$	using Theorem 1
	→ $\int_{0}^{w}\frac{{\mathrm{ID}}_{F}^{*}\;F_{w}\left(t\right)}{t}\ln\frac{{\mathrm{ID}}_{F}^{*}\;F_{w}\left(t\right)}{{\mathrm{ID}}_{G}^{*}\;G_{w}\left(t\right)}\;dt$	using Lemma 3
	→ $\int_{0}^{w}\frac{{\mathrm{ID}}_{F}^{*}}{t}{\left(\frac{t}{w}\right)}^{{\mathrm{ID}}_{F}^{*}}\ln\left[\frac{{\mathrm{ID}}_{F}^{*}}{{\mathrm{ID}}_{G}^{*}}{\left(\frac{t}{w}\right)}^{{\mathrm{ID}}_{F}^{*}-{\mathrm{ID}}_{G}^{*}}\right]\;dt$	using Lemma 1
	→ ρ−lnρ−1	$\rho =\frac{{\mathrm{ID}}_{G}^{*}}{{\mathrm{ID}}_{F}^{*}}$
JS Divergence	JS(F;G,w) → $\frac{1}{2}\left(\mathrm{KL}(F;M,w)+\mathrm{KL}(G;M,w)\right)$	$M\left(t\right)=\frac{1}{2}\left(F(t)+G(t)\right)$
	→ $\frac{1}{2}\left(\frac{{\mathrm{ID}}_{M}^{*}}{{\mathrm{ID}}_{F}^{*}}-\ln\frac{{\mathrm{ID}}_{M}^{*}}{{\mathrm{ID}}_{F}^{*}}-1+\frac{{\mathrm{ID}}_{M}^{*}}{{\mathrm{ID}}_{G}^{*}}-\ln\frac{{\mathrm{ID}}_{M}^{*}}{{\mathrm{ID}}_{G}^{*}}-1\right)$	${\mathrm{ID}}_{M}^{*}=\min\left\{{\mathrm{ID}}_{F}^{*},{\mathrm{ID}}_{G}^{*}\right\}$
	→ $\frac{1}{2}\left(\frac{{\mathrm{ID}}_{M}^{*}}{B}+\frac{{\mathrm{ID}}_{M}^{*}}{{\mathrm{ID}}_{M}^{*}}-\ln\frac{{\mathrm{ID}}_{M}^{*}}{B}-\ln\frac{{\mathrm{ID}}_{M}^{*}}{{\mathrm{ID}}_{M}^{*}}-2\right)$	let $B=\max\{{\mathrm{ID}}_{F}^{*},{\mathrm{ID}}_{G}^{*}\}$
	→ $\frac{1}{2}\left(\tau -\ln\tau -1\right)$	$\tau =\min\left\{\frac{{\mathrm{ID}}_{G}^{*}}{{\mathrm{ID}}_{F}^{*}},\frac{{\mathrm{ID}}_{F}^{*}}{{\mathrm{ID}}_{G}^{*}}\right\}$

**Table 4:** Derivations of asymptotic relationships between tail distances and local intrinsic dimensionality. Each step shows the equivalences between the formulations when *w* is allowed to tend to zero. In the comments column, for each step of the derivation, the lemmas invoked are stated, as well as any additional assumptions made. For each tail distance, the first step of the derivations shows an expansion by which the monotonicity of each factor can be verified. If a normalization or weighting is needed, the details are shown in a comment in the final step. In all cases, *F* and *G* are assumed to be smooth growth functions.

Tail Measure	Derivation Steps	Comments
L2 Distance	L2D(F;G,w) → $\int_{0}^{w}{\left(F_{w}^{\prime }\left(t\right)-G_{w}^{\prime }\left(t\right)\right)}^{2}\;dt$	
	→ $\int_{0}^{w}{\left(\frac{{\mathrm{ID}}_{F}\left(t\right)\;F_{w}\left(t\right)}{t}-\frac{{\mathrm{ID}}_{G}\left(t\right)\;G_{w}\left(t\right)}{t}\right)}^{2}\;dt$	using Theorem 1
	→ $\int_{0}^{w}{\left(\frac{{\mathrm{ID}}_{F}^{*}\;F_{w}\left(t\right)}{t}\right)}^{2}-2\frac{{\mathrm{ID}}_{F}^{*}\;F_{w}\left(t\right)}{t}\cdot \frac{{\mathrm{ID}}_{G}^{*}\;G_{w}\left(t\right)}{t}+{\left(\frac{{\mathrm{ID}}_{G}^{*}\;G_{w}\left(t\right)}{t}\right)}^{2}\;dt$	using Lemma 3
	→ $\int_{0}^{w}\frac{{\left({\mathrm{ID}}_{F}^{*}\right)}^{2}}{t^{2}}{\left(\frac{t}{w}\right)}^{2{\mathrm{ID}}_{F}^{*}}-\frac{2{\mathrm{ID}}_{F}^{*}{\mathrm{ID}}_{G}^{*}}{t^{2}}{\left(\frac{t}{w}\right)}^{{\mathrm{ID}}_{F}^{*}+{\mathrm{ID}}_{G}^{*}}+\frac{{\left({\mathrm{ID}}_{G}^{*}\right)}^{2}}{t^{2}}{\left(\frac{t}{w}\right)}^{2{\mathrm{ID}}_{G}^{*}}\;dt$	using Lemma 1
	→ $\frac{1}{w}\cdot \frac{{\left({\mathrm{ID}}_{F}^{*}-{\mathrm{ID}}_{G}^{*}\right)}^{2}}{2({\mathrm{ID}}_{F}^{*}+{\mathrm{ID}}_{G}^{*}-1)}\left[1+\frac{1}{\left(2{\mathrm{ID}}_{F}^{*}-1\right)\left(2{\mathrm{ID}}_{G}^{*}-1\right)}\right]$	weight by *w*
Hellinger Distance	HD(F;G,w) → $\sqrt{\frac{1}{2}\int_{0}^{w}{\left(\sqrt{F_{w}^{\prime }\left(t\right)}-\sqrt{G_{w}^{\prime }\left(t\right)}\right)}^{2}\;dt}$	
	→ $\sqrt{\frac{1}{2}\int_{0}^{w}{\left(\sqrt{\frac{{\mathrm{ID}}_{F}\left(t\right)\;F_{w}\left(t\right)}{t}}-\sqrt{\frac{{\mathrm{ID}}_{G}\left(t\right)\;G_{w}\left(t\right)}{t}}\right)}^{2}\;dt}$	using Theorem 1
	→ $\sqrt{\frac{1}{2}\int_{0}^{w}\frac{{\mathrm{ID}}_{F}^{*}\;F_{w}\left(t\right)}{t}-2\frac{\sqrt{{\mathrm{ID}}_{F}^{*}\;F_{w}\left(t\right)\cdot {\mathrm{ID}}_{G}^{*}\;G_{w}\left(t\right)}}{t}+\frac{{\mathrm{ID}}_{G}^{*}\;G_{w}\left(t\right)}{t}\;dt}$	using Lemma 3
	→ $\sqrt{\int_{0}^{w}\frac{1}{2t}\left({\mathrm{ID}}_{F}^{*}{\left(\frac{t}{w}\right)}^{{\mathrm{ID}}_{F}^{*}}-2\sqrt{{\mathrm{ID}}_{F}^{*}{\mathrm{ID}}_{G}^{*}}{\left(\frac{t}{w}\right)}^{({\mathrm{ID}}_{F}^{*}+{\mathrm{ID}}_{G}^{*})/2}+{\mathrm{ID}}_{G}^{*}{\left(\frac{t}{w}\right)}^{{\mathrm{ID}}_{G}^{*}}\right)\;dt}$	using Lemma 1
	→ $\frac{\left|1-\sqrt{\rho }\right|}{\sqrt{1+\rho }}$	$\rho =\frac{{\mathrm{ID}}_{G}^{*}}{{\mathrm{ID}}_{F}^{*}}$
$\chi^{2}$-Divergence	$\chi^{2}D\left(F;G,w\right)$ → $\int_{0}^{w}\frac{{\left(F_{w}^{\prime }\left(t\right)-G_{w}^{\prime }\left(t\right)\right)}^{2}}{G_{w}^{\prime }\left(t\right)}\;dt$	
	→ $\int_{0}^{w}{\left(\frac{{\mathrm{ID}}_{F}\left(t\right)\;F_{w}\left(t\right)}{t}-\frac{{\mathrm{ID}}_{G}\left(t\right)\;G_{w}\left(t\right)}{t}\right)}^{2}\frac{t}{{\mathrm{ID}}_{G}\left(t\right)\;G_{w}\left(t\right)}\;dt$	using Theorem 1
	→ $\int_{0}^{w}\left[{\left(\frac{{\mathrm{ID}}_{F}^{*}\;F_{w}\left(t\right)}{t}\right)}^{2}-2\frac{{\mathrm{ID}}_{F}^{*}\;F_{w}\left(t\right)}{t}\cdot \frac{{\mathrm{ID}}_{G}^{*}\;G_{w}\left(t\right)}{t}+{\left(\frac{{\mathrm{ID}}_{G}^{*}\;G_{w}\left(t\right)}{t}\right)}^{2}\right]\frac{t}{{\mathrm{ID}}_{G}^{*}\;G_{w}\left(t\right)}\;dt$	using Lemma 3
	→ $\int_{0}^{w}\left[\frac{{\left({\mathrm{ID}}_{F}^{*}\right)}^{2}}{t^{2}}{\left(\frac{t}{w}\right)}^{2{\mathrm{ID}}_{F}^{*}}-\frac{2{\mathrm{ID}}_{F}^{*}{\mathrm{ID}}_{G}^{*}}{t^{2}}{\left(\frac{t}{w}\right)}^{{\mathrm{ID}}_{F}^{*}+{\mathrm{ID}}_{G}^{*}}+\frac{{\left({\mathrm{ID}}_{G}^{*}\right)}^{2}}{t^{2}}{\left(\frac{t}{w}\right)}^{2{\mathrm{ID}}_{G}^{*}}\right]\frac{t}{{\mathrm{ID}}_{G}^{*}}{\left(\frac{w}{t}\right)}^{{\mathrm{ID}}_{G}^{*}}\;dt$	using Lemma 1
	→ $\frac{{\left(1-\rho \right)}^{2}}{\rho (2-\rho )}$	$\rho =\frac{{\mathrm{ID}}_{G}^{*}}{{\mathrm{ID}}_{F}^{*}}$
α-Divergence	αD(F;G,w) → $\frac{1}{\alpha (1-\alpha )}\int_{0}^{w}\alpha F_{w}^{\prime }\left(t\right)+\left(1-\alpha \right)G_{w}^{\prime }\left(t\right)-{\left(F_{w}^{\prime }\left(t\right)\right)}^{\alpha }{\left(G_{w}^{\prime }\left(t\right)\right)}^{1-\alpha }\;dt$	
	→ $\frac{1}{\alpha (1-\alpha )}\int_{0}^{w}\frac{\alpha {\mathrm{ID}}_{F}\left(t\right)\;F_{w}\left(t\right)}{t}+\frac{\left(1-\alpha \right){\mathrm{ID}}_{G}\left(t\right)\;G_{w}\left(t\right)}{t}-{\left(\frac{{\mathrm{ID}}_{F}\left(t\right)\;F_{w}\left(t\right)}{t}\right)}^{\alpha }{\left(\frac{{\mathrm{ID}}_{G}\left(t\right)\;G_{w}\left(t\right)}{t}\right)}^{1-\alpha }\;dt$	using Theorem 1
	→ $\frac{1}{\alpha (1-\alpha )}\int_{0}^{w}\frac{\alpha {\mathrm{ID}}_{F}^{*}\;F_{w}\left(t\right)}{t}+\frac{\left(1-\alpha \right){\mathrm{ID}}_{G}^{*}\;G_{w}\left(t\right)}{t}-{\left(\frac{{\mathrm{ID}}_{F}^{*}\;F_{w}\left(t\right)}{t}\right)}^{\alpha }{\left(\frac{{\mathrm{ID}}_{G}^{*}\;G_{w}\left(t\right)}{t}\right)}^{1-\alpha }\;dt$	using Lemma 3
	→ $\frac{1}{\alpha (1-\alpha )}\int_{0}^{w}\frac{\alpha {\mathrm{ID}}_{F}^{*}}{t}{\left(\frac{t}{w}\right)}^{{\mathrm{ID}}_{F}^{*}}+\frac{\left(1-\alpha \right){\mathrm{ID}}_{G}^{*}}{t}{\left(\frac{t}{w}\right)}^{{\mathrm{ID}}_{G}^{*}}-\frac{{\left({\mathrm{ID}}_{F}^{*}\right)}^{\alpha }{\left({\mathrm{ID}}_{G}^{*}\right)}^{1-\alpha }}{t}{\left(\frac{t}{w}\right)}^{\alpha {\mathrm{ID}}_{F}^{*}+\left(1-\alpha \right){\mathrm{ID}}_{G}^{*}}\;dt$	using Lemma 1
	→ $\frac{1}{\alpha (1-\alpha )}\left(1-\frac{{\left({\mathrm{ID}}_{F}^{*}\right)}^{\alpha }{\left({\mathrm{ID}}_{G}^{*}\right)}^{1-\alpha }}{\alpha {\mathrm{ID}}_{F}^{*}+\left(1-\alpha \right){\mathrm{ID}}_{G}^{*}}\right)$	
	→ $\frac{1}{\alpha (1-\alpha )}\left(1-\frac{1}{\alpha \rho^{\alpha -1}+\left(1-\alpha \right)\rho^{\alpha }}\right)$	$\rho =\frac{{\mathrm{ID}}_{G}^{*}}{{\mathrm{ID}}_{F}^{*}}$

**Table 5:** Derivations of asymptotic relationships between tail Wasserstein distances and local intrinsic dimensionality. Each step shows the equivalences between the formulations when *w* is allowed to tend to zero. In the comments column, for each step of the derivation, the lemmas invoked are stated, as well as any additional assumptions made. Normalization details are shown in a comment in the final step. In all cases, *F* and *G* are assumed to be invertible smooth growth functions.

Tail Measure	Derivation Steps	Comments
Wasserstein Distance	${\mathrm{WD}}_{2}\left(F;G,w\right)$ → $\sqrt{\int_{0}^{1}{\left(F_{w}^{-1}\left(u\right)-G_{w}^{-1}\left(u\right)\right)}^{2}\;du}$	
	→ $\sqrt{\int_{0}^{1}{\left(F_{w}^{-1}\left(u\right)\right)}^{2}-2F_{w}^{-1}\left(u\right)\cdot G_{w}^{-1}\left(u\right)+{\left(G_{w}^{-1}\left(u\right)\right)}^{2}\;du}$	
p=2	→ $\sqrt{\int_{0}^{1}w^{2}u^{\frac{2}{{\mathrm{ID}}_{F}^{*}}}-2w^{2}u^{\frac{1}{{\mathrm{ID}}_{F}^{*}}+\frac{1}{{\mathrm{ID}}_{G}^{*}}}+w^{2}u^{\frac{2}{{\mathrm{ID}}_{G}^{*}}}\;du}$	using Lemma 2
	→ $w\sqrt{\frac{1}{\frac{2}{{\mathrm{ID}}_{F}^{*}}+1}-\frac{2}{\frac{1}{{\mathrm{ID}}_{F}^{*}}+\frac{1}{{\mathrm{ID}}_{G}^{*}}+1}+\frac{1}{\frac{2}{{\mathrm{ID}}_{G}^{*}}+1}}$	weight by $\frac{1}{w}$
Wasserstein Distance	${\mathrm{WD}}_{p}\left(F;G,w\right)$ → ${\left(\int_{0}^{1}{\left(F_{w}^{-1}\left(u\right)-G_{w}^{-1}\left(u\right)\right)}^{p}\;du\right)}^{\frac{1}{p}}$	
p∈N, *p* even	→ ${\left(\int_{0}^{1}\sum_{j=0}^{p}{\left(-1\right)}^{j}\left(\overset{p}{j}\right){\left(F_{w}^{-1}\left(u\right)\right)}^{p-j}{\left(G_{w}^{-1}\left(u\right)\right)}^{j}\;du\right)}^{\frac{1}{p}}$	
	→ ${\left(\int_{0}^{1}\sum_{j=0}^{p}{\left(-1\right)}^{j}\left(\overset{p}{j}\right){\left(wu^{\frac{1}{{\mathrm{ID}}_{F}^{*}}}\right)}^{p-j}{\left(wu^{\frac{1}{{\mathrm{ID}}_{G}^{*}}}\right)}^{j}\;du\right)}^{\frac{1}{p}}$	using Lemma 2
	→ $w{\left(\sum_{j=0}^{p}\frac{{\left(-1\right)}^{j}\left(\overset{p}{j}\right)}{\left(p-j\right)\cdot {\left({\mathrm{ID}}_{F}^{*}\right)}^{-1}+j\cdot {\left({\mathrm{ID}}_{G}^{*}\right)}^{-1}+1}\right)}^{\frac{1}{p}}$	weight by $\frac{1}{w}$

**Table 6:** Asymptotic equivalences between LID formulations and tail measures of entropy or divergence for locally spherically symmetric distributions in the *n*-dimensional Euclidean setting. In each case, the density functions are assumed to be *f* and *g*, and the CDFs *F* and *G* of their induced distance distributions are assumed to be smooth growth functions. In the results, $V_{n}\left(r\right)$ and $S_{n-1}\left(r\right)$ denote the volume and surface area of the *n*-dimensional ball with radius *r* (respectively). In some cases, for the asymptotic limit to exist non-trivially (that is, to be both finite and non-zero), the tail entropy or tail divergence must be normalized by some multiplicative factor dependent on the tail volume $V_{n}\left(w\right)$.

Tail Measure	Formulation	Limit as $w\to 0^{+}$
Entropy	H(f,w) = $-\int_{B(w)}f_{w}\ln f_{w}\;dB\left(w\right)\;=\;-\int_{0}^{w}F_{w}^{\prime }\left(t\right)\ln\frac{F_{w}^{\prime }\left(t\right)}{S_{n-1}\left(t\right)}\;dt$	Diverges (no reweighting possible)
Varentropy	VarH(f,w) = $\int_{B(w)}f_{w}\;\ln^{2}f_{w}\;dB\left(w\right)-{\left(\int_{B(w)}f_{w}\ln f_{w}\;dB\left(w\right)\right)}^{2}$	${\left(1-\frac{1}{\varphi }\right)}^{2}$
	= $\int_{0}^{w}F_{w}^{\prime }\left(t\right)\ln^{2}\frac{F_{w}^{\prime }\left(t\right)}{S_{n-1}\left(t\right)}\;dt\;-{\left(\int_{0}^{w}F_{w}^{\prime }\left(t\right)\ln\frac{F_{w}^{\prime }\left(t\right)}{S_{n-1}\left(t\right)}\;dt\right)}^{2}$	$\varphi =\frac{{\mathrm{ID}}_{F}^{*}}{n}$
*q*-Entropy	$H_{q}\left(f,w\right)$ = $\frac{1}{q-1}\int_{B(w)}f_{w}-f_{w}^{q}\;dB\left(w\right)$	$\frac{1}{q-1}$ if q<1
	= $\frac{1}{q-1}\int_{0}^{w}F_{w}^{\prime }\left(t\right)-\frac{{\left(F_{w}^{\prime }\left(t\right)\right)}^{q}}{{\left(S_{n-1}\left(t\right)\right)}^{q-1}}\;dt$	diverges if q>1
Normalized	$\frac{1}{V_{n}\left(w\right)}\mathrm{cH}\left(f,w\right)$ = $-\frac{1}{V_{n}\left(w\right)}\int_{x\in B(w)}F_{w}\left(∥x∥\right)\;\ln F_{w}\left(∥x∥\right)\;dB\left(w\right)$	$\frac{\varphi }{{\left(\varphi +1\right)}^{2}}$
Cumulative Entropy	= $-\frac{1}{V_{n}\left(w\right)}\int_{0}^{w}\left(F_{w}\left(t\right)\;\ln F_{w}\left(t\right)\right)\cdot S_{n-1}\left(t\right)\;dt$	$\varphi =\frac{{\mathrm{ID}}_{F}^{*}}{n}$
Normalized	$\frac{1}{V_{n}\left(w\right)}{\mathrm{cH}}_{q}\left(f,w\right)$ = $-\frac{1}{V_{n}\left(w\right)}\cdot \frac{1}{q-1}\int_{x\in B(w)}F_{w}\left(∥x∥\right)-{\left(F_{w}\left(∥x∥\right)\right)}^{q}\;dB\left(w\right)$	$\frac{\varphi }{\left(q\varphi +1)(\varphi +1\right)}$ if q≠1
Cumulative *q*-Entropy	= $\frac{1}{V_{n}\left(w\right)}\cdot \frac{1}{q-1}\int_{0}^{w}\left(F_{w}\left(t\right)-{\left(F_{w}\left(t\right)\right)}^{q}\right)\cdot S_{n-1}\left(t\right)\;dt$	$\varphi =\frac{{\mathrm{ID}}_{F}^{*}}{n}$
Normalized Entropy Power	$\frac{1}{V_{n}\left(w\right)}\mathrm{HP}\left(f,w\right)$ = $\frac{1}{V_{n}\left(w\right)}\exp\left(H(f,w)\right)$	$\frac{1}{\varphi }\exp\left(1-\frac{1}{\varphi }\right);\;\;\;\varphi =\frac{{\mathrm{ID}}_{F}^{*}}{n}$
Normalized *q*-Entropy Power	$\frac{1}{V_{n}\left(w\right)}{\mathrm{HP}}_{q}\left(f,w\right)$ = $\frac{1}{V_{n}\left(w\right)}{\left[1+\left(1-q\right)\;H_{q}\left(f,w\right)\right]}^{\frac{1}{1-q}}$	${\left(\frac{\varphi^{q}}{q\varphi -q+1}\right)}^{\frac{1}{1-q}}$ ; $\varphi =\frac{{\mathrm{ID}}_{F}^{*}}{n}$
		if q≠1 and qφ−q+1>0
Cross Entropy	XH(f;g,w) = $-\int_{B(w)}f_{w}\ln g_{w}\;dB\left(w\right)=-\int_{0}^{w}F_{w}^{\prime }\left(t\right)\ln\frac{G_{w}^{\prime }\left(t\right)}{S_{n-1}\left(t\right)}\;dt$	Diverges (no reweighting possible)
Normalized Cross Entropy Power	$\frac{1}{V_{n}\left(w\right)}\mathrm{XHP}\left(f;g,w\right)$ = $\frac{1}{V_{n}\left(w\right)}\exp\left(\mathrm{XH}(f;g,w)\right)$	$\frac{1}{\gamma }\exp\left(\frac{\gamma -1}{\varphi }\right);\;\;\;\varphi =\frac{{\mathrm{ID}}_{F}^{*}}{n}\;;\;\gamma =\frac{{\mathrm{ID}}_{G}^{*}}{n}$
Weighted	$V_{n}\left(w\right)\cdot L2D\left(f;g,w\right)$ = $V_{n}\left(w\right)\;\int_{B(w)}{\left(f_{w}-g_{w}\right)}^{2}\;dB\left(w\right)$	$\frac{{\left(\varphi -\gamma \right)}^{2}}{2(\varphi +\gamma -1)}\left[1+\frac{1}{\left(2\varphi -1)(2\gamma -1\right)}\right]$
L2 Distance	= $V_{n}\left(w\right)\int_{0}^{w}\frac{1}{S_{n-1}\left(t\right)}{\left(F_{w}^{\prime }\left(t\right)-G_{w}^{\prime }\left(t\right)\right)}^{2}\;dt$	$\varphi =\frac{{\mathrm{ID}}_{F}^{*}}{n}\;;\;\gamma =\frac{{\mathrm{ID}}_{G}^{*}}{n}$
		${\mathrm{ID}}_{F}^{*}>\frac{1}{2}$; ${\mathrm{ID}}_{G}^{*}>\frac{1}{2}$
Hellinger Distance	HD(f;g,w) = $\sqrt{\frac{1}{2}\int_{B(w)}{\left(\sqrt{f_{w}}-\sqrt{g_{w}}\right)}^{2}\;dB\left(w\right)}$	$\frac{\left|1-\sqrt{\rho }\right|}{\sqrt{1+\rho }}$
	= $\sqrt{\frac{1}{2}\int_{0}^{w}{\left(\sqrt{F_{w}^{\prime }\left(t\right)}-\sqrt{G_{w}^{\prime }\left(t\right)}\right)}^{2}\;dt}$	$\rho =\frac{{\mathrm{ID}}_{G}^{*}}{{\mathrm{ID}}_{F}^{*}}$
$\chi^{2}$-Divergence	$\chi^{2}D\left(f;g,w\right)$ = $\int_{B(w)}\frac{{\left(f_{w}-g_{w}\right)}^{2}}{g_{w}}\;dB\left(w\right)$	$\frac{{\left(1-\rho \right)}^{2}}{\rho (2-\rho )}$
	= $\int_{0}^{w}\frac{{\left(F_{w}^{\prime }\left(t\right)-G_{w}^{\prime }\left(t\right)\right)}^{2}}{G_{w}^{\prime }\left(t\right)}\;dt$	$\rho =\frac{{\mathrm{ID}}_{G}^{*}}{{\mathrm{ID}}_{F}^{*}}\;;\;\rho <2$
α-Divergence	αD(f;g,w) = $\frac{1}{\alpha (1-\alpha )}\int_{B(w)}\alpha f_{w}+\left(1-\alpha \right)g_{w}-f_{w}^{\alpha }g_{w}^{1-\alpha }\;dB\left(w\right)$	$\frac{1}{\alpha (1-\alpha )}\left(1-\frac{1}{\alpha \rho^{\alpha -1}+\left(1-\alpha \right)\rho^{\alpha }}\right)$
	= $\frac{1}{\alpha (1-\alpha )}\int_{0}^{w}\alpha F_{w}^{\prime }\left(t\right)+\left(1-\alpha \right)G_{w}^{\prime }\left(t\right)$	$\rho =\frac{{\mathrm{ID}}_{G}^{*}}{{\mathrm{ID}}_{F}^{*}}$
	$\;-{\left(F_{w}^{\prime }\left(t\right)\right)}^{\alpha }{\left(G_{w}^{\prime }\left(t\right)\right)}^{1-\alpha }\;dt$	Require α+ρ(1−α)>0
KL Divergence	KL(f;g,w) = $\int_{B(w)}f_{w}\ln\frac{f_{w}}{g_{w}}\;dB\left(w\right)\;=\;\int_{0}^{w}F_{w}^{\prime }\left(t\right)\ln\frac{F_{w}^{\prime }\left(t\right)}{G_{w}^{\prime }\left(t\right)}\;dt$	$\rho -\ln\rho -1;\;\;\;\rho =\frac{{\mathrm{ID}}_{G}^{*}}{{\mathrm{ID}}_{F}^{*}}$
JS Divergence	JS(f;g,w) = $\frac{1}{2}\left(\mathrm{KL}\left(f;\frac{f+g}{2},w\right)+\mathrm{KL}\left(g;\frac{f+g}{2},w\right)\right)$	$\frac{\tau -\ln\tau -1}{2};\;\;\tau =\min\left\{\rho ,\frac{1}{\rho }\right\};\;\;\rho =\frac{{\mathrm{ID}}_{G}^{*}}{{\mathrm{ID}}_{F}^{*}}$

## Data Availability

Not applicable.
